# Attenuation of Native Hyperpolarization-Activated, Cyclic Nucleotide-Gated Channel Function by the Volatile Anesthetic Sevoflurane in Mouse Thalamocortical Relay Neurons

**DOI:** 10.3389/fncel.2020.606687

**Published:** 2021-01-21

**Authors:** Stefan Schwerin, Claudia Kopp, Elisabeth Pircher, Gerhard Schneider, Matthias Kreuzer, Rainer Haseneder, Stephan Kratzer

**Affiliations:** Department of Anesthesiology and Intensive Care Medicine, Technical University of Munich School of Medicine, Munich, Germany

**Keywords:** sevoflurane, thalamus, thalamocortical relay neurons, HCN channel, mechanisms of anesthesia, patch-clamp

## Abstract

As thalamocortical relay neurons are ascribed a crucial role in signal propagation and information processing, they have attracted considerable attention as potential targets for anesthetic modulation. In this study, we analyzed the effects of different concentrations of sevoflurane on the excitability of thalamocortical relay neurons and hyperpolarization-activated, cyclic-nucleotide gated (HCN) channels, which play a decisive role in regulating membrane properties and rhythmic oscillatory activity. The effects of sevoflurane on single-cell excitability and native HCN channels were investigated in acutely prepared brain slices from adult wild-type mice with the whole-cell patch-clamp technique, using voltage-clamp and current-clamp protocols. Sevoflurane dose-dependently depressed membrane biophysics and HCN-mediated parameters of neuronal excitability. Respective half-maximal inhibitory and effective concentrations ranged between 0.30 (95% CI, 0.18–0.50) mM and 0.88 (95% CI, 0.40–2.20) mM. We witnessed a pronounced reduction of HCN dependent I_h_ current amplitude starting at a concentration of 0.45 mM [relative change at −133 mV; 0.45 mM sevoflurane: 0.85 (interquartile range, 0.79–0.92), *n* = 12, *p* = 0.011; 1.47 mM sevoflurane: 0.37 (interquartile range, 0.34–0.62), *n* = 5, *p* < 0.001] with a half-maximal inhibitory concentration of 0.88 (95% CI, 0.40–2.20) mM. In contrast, effects on voltage-dependent channel gating were modest with significant changes only occurring at 1.47 mM [absolute change of half-maximal activation potential; 1.47 mM: −7.2 (interquartile range, −10.3 to −5.8) mV, *n* = 5, *p* = 0.020]. In this study, we demonstrate that sevoflurane inhibits the excitability of thalamocortical relay neurons in a concentration-dependent manner within a clinically relevant range. Especially concerning its effects on native HCN channel function, our findings indicate substance-specific differences in comparison to other anesthetic agents. Considering the importance of HCN channels, the observed effects might mechanistically contribute to the hypnotic properties of sevoflurane.

## Introduction

Since its introduction into clinical practice, the volatile anesthetic sevoflurane has been enjoying popularity worldwide with several studies demonstrating its safe and efficient use (Brioni et al., 2017). There is still an ongoing debate over the exact mechanisms of how general anesthetics like sevoflurane induce a reversible loss of consciousness. One of the key questions is which anatomical sites or neuronal networks present the primary targets of anesthetic action. So-called “top-down” theories emphasize disruptive effects of general anesthetics on functional connectivity in cortical networks, whereas “bottom-up” approaches focus on hierarchical processing of sensory information (Mashour, 2014). In this context, the thalamus as part of the intricately interconnected thalamocortical network (McCormick and Bal, 1997) as well as ascending subcortical pathways (Guillery and Sherman, 2011) has attracted considerable attention. As most pharmaceutical agents with hypnotic properties cause depression of thalamic metabolism (Alkire and Miller, 2005), the thalamus was initially thought to serve as a “consciousness switch” *via* hyperpolarization blockade of thalamocortical neurons (Alkire et al., 2000). More recent studies, which suggest that the anesthetic-induced loss of consciousness cannot sufficiently be explained by the interruption of the thalamocortical feed-forward relay of peripheral input to sensory cortices alone, have evolved our understanding of thalamic function. The thalamus serves as an integration hub for cortical communication as well as multimodal information processing. Preserved thalamocortical connectivity between higher-order nuclei and frontal-parietal networks seems a likely prerequisite for maintaining consciousness (Liu et al., 2013; Mashour, 2014; Ranft et al., 2016; Sherman, 2016).

Converging observed macro effects of anesthetic action onto a neuronal level, the importance of thalamocortical neurons in regulating activity within the thalamocortical network is well established. They display two modes of action. A shift of the resting membrane potential towards hyperpolarization terminates tonic single-spike activity and facilitates rhythmic burst firing that leads to synchronized oscillations within the thalamocortical network (Franks, 2008). Subsequent changes in electroencephalogram recordings are typically associated with forms of slow-wave sleep (McCormick and Bal, 1997). Intriguingly, there is evidence linking the initiation of both natural sleep and anesthetic-induced loss of consciousness to the thalamus (Baker et al., 2014). Excitability of thalamocortical neurons and their ability to act as cellular pacemakers for oscillations depends on the inwardly directed, mixed-cation current I_h_ (Destexhe and Sejnowski, 2003) that is mediated by special members of the voltage-gated potassium channel superfamily, so-called hyperpolarization-activated, cyclic-nucleotide-gated (HCN) channels (Biel et al., 2009). In the case of thalamocortical relay neurons, HCN2 (Ludwig et al., 2003) and, more recently, HCN4 (Zobeiri et al., 2019) were described as the predominant isoforms to mediate I_h_ and to substantially modulate firing activity. HCN channels have emerged as a promising molecular target of anesthetic action over the past decades (Goldstein, 2015; Riegelhaupt et al., 2018). Several anesthetics inhibit HCN channel function in thalamocortical relay neurons *in vitro*, including propofol (Ying et al., 2006), pentobarbital (Wan et al., 2003), xenon (Mattusch et al., 2015), and halothane (Chen et al., 2005; Budde et al., 2008). Sevoflurane acts on a wide range of molecular targets (Hapfelmeier et al., 2001; Rudolph and Antkowiak, 2004). However, its effect on native HCN channel function in thalamocortical relay neurons remains poorly understood. Available electrophysiological data are either exploratory in nature (Budde et al., 2008) or stem from different neuronal cell populations (Sirois et al., 1998; Sugasawa et al., 2018).

In our experiments, we investigated the effects of clinically relevant sevoflurane concentrations on I_h_ and HCN-mediated excitability of thalamocortical relay neurons. For this purpose, we used the patch-clamp technique in acute brain slices from mice.

## Materials and Methods

### Thalamocortical Slice Preparation

Experimental protocols as described were approved by the Ethical Committee on Animal Care and Use of the Government of Bavaria (Munich, Germany). Brains were surgically removed from female C57Bl/6N mice (P28–P35) under isoflurane anesthesia. Brain slices (350 μm thick) containing the ventrobasal (VB) complex of the thalamus with preserved thalamocortical connectivity were prepared using a vibratome (HM 650 V, Thermo Fisher Scientific, Walldorf, Germany) according to Agmon and Connors (1991). Slice preparation was conducted in ice-cold artificial cerebrospinal fluid [aCSF, containing (in mM): NaCl, 125; KCl, 2.5; NaH_2_PO_4_, 1.25; D-glucose, 25; NaHCO_3_, 25; MgCl_2_, 6; CaCl_2_, 0.5; pH: 7.4] saturated with carbogen (95% O_2_/5% CO_2_). Following preparation, all slices were incubated in a storage chamber for at least 30 min at 34°C using a standard aCSF [(in mM): NaCl, 125; KCl, 2.5; NaH_2_PO_4_, 1.25; D-glucose, 25; NaHCO_3_, 25; MgCl_2_, 1; CaCl_2_, 2; pH: 7.4]. Slices were then allowed to recover for at least another 30 min in carbogenated standard aCSF at room temperature (20–24°C).

### Application of the Anesthetic Sevoflurane

Under control conditions, the slices were kept in standard aCSF solely aerated with carbogen (0 mM sevoflurane). Following the establishment of stable baseline recordings, sevoflurane was added to the perfusate by passing the carbogen through a calibrated agent-specific vaporizer (Drägerwerk, Lübeck, Germany) before aerating the circulating aCSF with it, as previously described (Kratzer et al., 2017). We applied sevoflurane at five different concentrations with the corresponding vaporizer dial settings (0.4, 1.3, 1.8, 3.2, and 8.0 vol.%). During the experiments, concentrations were continuously monitored using a Capnomac Ultima (Datex Ohmeda, Duisburg, Germany). To allow for sufficient equilibration of sevoflurane, we waited 20 min for each concentration before recordings. Different concentrations of sevoflurane were examined in different brain slices for each experiment. To account for potential concentration losses of sevoflurane due to evaporation in the open recording chamber, the application of sevoflurane *via* vaporizer was given preference instead of preparing and diluting sevoflurane stock solutions into the circulating aCSF. To further verify continuously stable concentrations in the recording chamber, corresponding aqueous concentrations of aCSF saturated with 0.6 vol.% (0.13 mM), 1.6 vol.% (0.45 mM), and 3.2 vol.% (0.69 mM) sevoflurane were obtained using headspace gas chromatography (Intertek AG, Schlieren, Switzerland). Concentrations of dissolved sevoflurane after saturation with 0.4 vol.% (0.08 mM), 1.3 vol.% (0.33 mM), 1.8 vol.% (0.45 mM) and 8.0 vol.% (1.47 mM) were then extrapolated from the measured values. These results are in good accordance with our previous findings which demonstrated a positive correlation between aqueous concentrations of sevoflurane with applied vapor dial settings (Haseneder et al., 2009). Sevoflurane was purchased from AbbVie (Ludwigshafen, Germany).

In the recording chamber of the patch-clamp setup, slices were continuously perfused with carbogenated standard aCSF at a flow rate of 5–8 ml/min. We used infrared videomicroscopy (Zeiss, Oberkochen, Germany) to visualize thalamocortical relay neurons in the VB thalamus. As described elsewhere (Ying et al., 2006), these neurons display a depolarizing voltage sag and an afterdepolarization potential in response to hyperpolarizing current steps which allow for their electrophysiological identification. Whole-cell recordings of single cells were performed using pipettes with an open tip resistance of 4–6 MΩ. Pipettes were filled with an intracellular solution comprised of (in mM): K-D-gluconate, 130; NaCl, 5; MgCl_2_, 2; HEPES, 10; EGTA, 0.5; K_2_-ATP, 2; Na_2_-GTP, 0.3; pH: 7.25.

### Electrophysiology

Whole-cell patch-clamp recordings were acquired using a discontinuous single-electrode voltage-clamp amplifier (SEC 10L; NPI Electronic, Tamm, Germany) with switching frequencies of 60–80 kHz (25% duty cycle). We continuously monitored series resistance. I_h_ currents were activated in the voltage-clamp mode through hyperpolarizing steps. Starting from −43 mV, the holding potential was incrementally hyperpolarized up to −133 mV by 10 mV steps. To ensure the stability of the whole-cell recordings and accounting for the acceleration of I_h_ activation kinetics with increasing hyperpolarization (Ludwig et al., 1999), the pulse length was reduced by 500 ms for every hyperpolarizing step (2.0 s pulse length at −133 mV; Meuth et al., 2006; Budde et al., 2008). As I_h_ reaches its maximum at membrane potentials beyond −110 mV (Pape, 1996), the maximal I_h_ current was determined from the −133 mV voltage step. Tail current amplitudes (I_tail_) were normalized to the tail current measured at the most hyperpolarized membrane potential (−133 mV) to assess steady-state activation. Voltage dependency of steady-state activation is well described by a Boltzmann distribution. To determine steady-state activation, the equation (I − I_min_/(I_max_ − I_min_) was used with I_max_ being the tail current amplitude for the voltage step from −133 mV to −103 mV and I_min_ for the voltage step from −43 mV to −103 mV. Fast (τ_fast_) and slow (τ_slow_) time constants were determined by fitting currents evoked during the hyperpolarizing step of −133 mV to a biexponential function, which represents time-dependent activation of I_h_ in thalamocortical relay neurons most accurately (Santoro et al., 2000).

**Figure 1:**
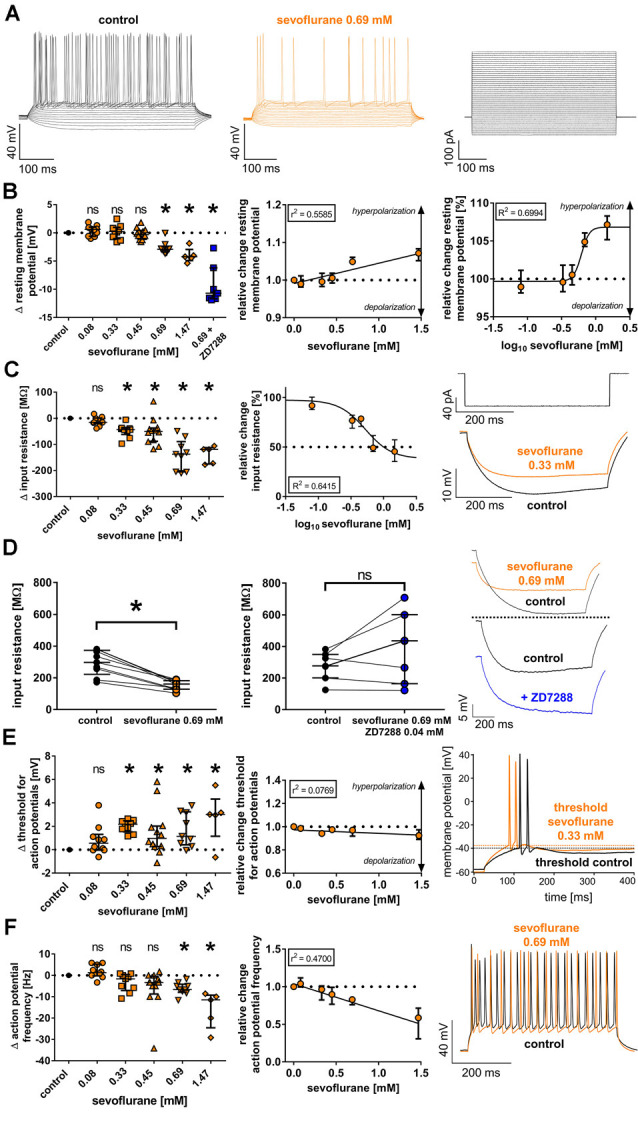
**(A)** Currents from −90 pA to +360 pA (10-pA steps) were injected into thalamocortical relay neurons and subsequent changes of the membrane potential were recorded under baseline conditions and in the presence of sevoflurane (representative current trace at 0.69 mM) in the current-clamp mode, with the corresponding current protocol depicted on the right. For clarity, only every second recording trace is depicted. **(B)** Sevoflurane significantly shifted the resting membrane potential [control: −56.9 (−58.7 to −55.6) mV, *n* = 45] towards hyperpolarization beginning at a concentration of 0.69 mM. This effect appeared to be concentration-dependent applying linear regression analysis and non-linear regression analysis (using a four-parameter logistic equation with sigmoidal curve fitting). Δ: absolute change compared to control. ns, not significant. *p < 0.05. log_10_: logarithmic scale of sevoflurane concentration (mM). **(C)** Sevoflurane significantly reduced neuronal input resistance [control: 227.6 (181.3–276.7) MΩ, *n* = 45] beginning at a concentration of 0.33 mM in a dose-dependent manner. The input resistance was measured by injecting a hyperpolarizing current (−90 pA). The current protocol with representative voltage traces under baseline conditions and in the presence of 0.33 mM sevoflurane is shown on the right. **(D)** While 0.69 mM sevoflurane led to a significant reduction of the neuronal input resistance, coapplying 0.04 mM of the selective HCN channel blocker ZD7288 reversed this effect. Representative voltage traces from both the sole application of 0.69 mM sevoflurane as well as in the presence of sevoflurane and 0.04 mM ZD7288 with respective baseline recordings are depicted on the right. **(E)** From a concentration of 0.33 mM on, sevoflurane significantly shifted the threshold for action potential firing [control: −38.9 (−40.1 to −36.0) mV, *n* = 45] towards depolarization. However, the linear regression analysis showed that this effect did not significantly correlate with the applied concentration. Representative voltage traces under control conditions and after application of 0.33 mM sevoflurane can be seen on the right, with the threshold for action potential firing being indicated by dotted lines. **(F)** In the presence of 0.69 mM and 1.47 mM, sevoflurane significantly decreased the frequency of tonic action potential firing [control: 39.2 (33.8–43.8) Hz, *n* = 45], which was induced by depolarizing current injections. Using linear regression analysis, the reduction appeared to be concentration-dependent. On the right, we show representative voltage traces of tonic action potential firing under control conditions and in the presence of 0.69 mM.

Current-clamp recordings were performed from the same thalamocortical neurons. By applying an intracellular current pulse (−350 pA, 500 ms), a depolarizing voltage sag of the membrane potential was elicited. We defined the sag amplitude as the difference between the peak hyperpolarization and the steady-state value. The rebound burst delay was determined as the time from the start of repolarization (the end of the hyperpolarizing current) to the peak of the first action potential. For each calculation, eight consecutive current-clamp recordings were graphically averaged using Igor Pro 5 (WaveMetrics, Lake Oswego, OR, USA). Electrical membrane properties were determined based on membrane voltage responses following current injections (−90 pA to +360 pA, 10 pA-increments, Figure 1A). Membrane potentials were not adjusted to control (pre-sevoflurane) levels for current-clamp recordings acquired after sevoflurane application.

We calculated the liquid junction potential based on intracellular and bath solutions with the help of the program Liquid Junction Potential Calculator (Clampex 10.7, Molecular Devices, San Jose, CA, USA) and performed online corrections. By adding 0.15 mM barium chloride to the standard aCSF for all recordings, inwardly rectifying potassium channels and subfamilies of the two-pore-domain acid-sensitive potassium channels were inhibited (Mattusch et al., 2015). Furthermore, barium contributes to demasking the voltage sag by significantly increasing its respective amplitude (Datunashvili et al., 2018). For a subset of experiments, the γ-aminobutyric acid type A (GABA_A_) receptor antagonist bicuculline methiodide (0.01 mM) or the HCN-channel antagonist ZD7288 [4-(N-Ethyl-N-phenylamino)-1,2 dimethyl-6-(methylamino) pyrimidinium chloride; 0.04 mM] were directly applied into the external recording solution. Salts and chemicals were purchased from Sigma–Aldrich (Steinheim, Germany), except for ZD7288 (Tocris Bioscience, Bristol, UK). All electrophysiological experiments were performed at room temperature (20–24°C).

The current responses were amplified, low-pass filtered (3 kHz), digitized (ITC-16 Mac computer interface, Instrutech Corp., Port Washington, WI, USA) with a sampling frequency of 9 kHz, and stored on a hard drive (Power Macintosh G3, Apple Computer Inc., Cupertino, CA, USA) with the data acquisition software Pulse version 8.5 (HEKA Elektronik, Lambrecht, Germany) without series resistance compensation. Cells were not included in the analysis if they met one of the following exclusion criteria: (1) no GΩ seal resistance; (2) initial resting membrane potential below −50 mV; (3) unstable series resistance or holding current (>20% change); and (4) no stable baseline conditions. Due to the nature of the performed experiments, the experimenter was not blinded to the experimental conditions.

### Statistical Analysis

For statistical analysis, we used GraphPad Prism 7.03 (GraphPad Software, San Diego, CA, USA). Sample sizes were chosen based on previous experience. Values are presented as median including the interquartile range if not stated otherwise.

Because of our limited sample size, we cannot sufficiently ensure normal distribution as well as homoscedasticity of the data. Upon visual inspection, our data often demonstrated a skewed distribution indicative of not normally distributed data. Therefore, we chose a more conservative approach with adequate robustness to outliers and applied non-parametrical tests with less statistical power. Wilcoxon matched-pairs signed-rank tests were used for simple comparisons and are indicated explicitly when applied. To test for statistically significant differences between applied sevoflurane concentrations and the baseline conditions of independent groups, we used the Kruskal–Wallis test and Dunn’s *post hoc* test to correct for multiple comparisons. Where applicable, *p*-values were adjusted for multiplicity, and differences were considered statistically significant when *p* < 0.05.

We applied linear regression analysis to test for the concentration dependence of observed effects and non-linear regression analysis using a four-parameter logistic equation with sigmoidal curve fitting to create dose-response relationships and to calculate half-maximal inhibitory concentrations (IC_50_) and half-maximal effective concentrations (EC_50_).

## Results

As we previously showed in a set of experiments using voltage-sensitive dye imaging techniques, the application of sevoflurane led to a dose-dependent, global reduction of cortical depolarization as well as to deferred thalamocortical signal propagation in response to stimulation of the VB thalamus (Kratzer et al., 2017). We hypothesized that these effects of sevoflurane might be attributable to a reduction of intrinsic neuronal excitability of thalamocortical relay neurons mediated by an impairment of HCN channel function, as tonic I_h_ is ascribed an important role in regulating membrane biophysics, like the resting membrane potential and the input resistance, as well as firing properties of neurons (He et al., 2014).

### Effects of Sevoflurane on Electrical Membrane Properties of VB Thalamocortical Relay Neurons

The resting membrane potential of thalamocortical relay neurons is determined by a set of ionic channels, including HCN channels (Meuth et al., 2006). In this study, we found a resting membrane potential of −56.9 mV (−58.7 to −55.6 mV, *n* = 45) under baseline conditions. At a concentration of 0.69 mM sevoflurane, we observed a significant shift of the resting membrane potential towards hyperpolarization, while lower concentrations did not change the resting membrane potential [0.08 mM: +0.6 (−0.6 to +1.0) mV, *n* = 10, *p* > 0.999; 0.33 mM: +0.2 (−1.0 to +1.0) mV, *n* = 9, *p* > 0.999; 0.45 mM: −0.3 (−1.1 to +0.4) mV, *n* = 12, *p* > 0.999; 0.69 mM: −2.9 (−3.4 to −2.4) mV, *n* = 9, *p* = 0.008; 1.47 mM: −4.2 (−4.9 to −3.0) mV, *n* = 5, *p* = 0.005, Figure 1B]. Applying linear regression analysis, the hyperpolarizing effect of sevoflurane on the resting membrane potential demonstrated a significant concentration dependence (*r*^2^ = 0.5585, *F* = 69.57, DF_n_, DF_d_ = 1.000, 55.00, *p* < 0.001, Figure 1B). Non-linear regression analysis using a four-parameter logistic equation with sigmoidal curve fitting yielded an EC_50_ of 0.62 (95% CI: 0.54–0.70) mM sevoflurane [Hill coefficient: +7.5 (95% CI: −2.4 to +12.7), *R*^2^ = 0.6994, Figure 1B]. We further tested the effect of coapplying the selective HCN channel blocker ZD7288 0.04 mM with 0.69 mM sevoflurane on the resting membrane potential. Under these conditions, the resting membrane potential was markedly stronger hyperpolarized compared to the effect of 0.69 mM sevoflurane alone with a median difference of −10.7 (−11.7 to −6.2) mV [control: −58.8 (−60.1 to −54.9) mV; 0.69 mM sevoflurane: −60.7 (−62.2 to −58.1) mV, *n* = 9, *p* = 0.004 vs. control: −60.3 (−61.9 to −59.2) mV; 0.69 mM sevoflurane + 0.04 mM ZD7288: −70.3 (−73.8 to −65.4) mV, *n* = 7, *p* = 0.016, Wilcoxon matched-pairs signed-rank test in each case].

HCN channel inhibition seems to increase membrane input resistance. By reducing a cell’s “leakiness,” an increased input resistance enhances the probability of excitatory postsynaptic currents to generate action potentials at synapses (Biel et al., 2009). To assess the effect of sevoflurane on the input resistance, we determined the voltage change following a hyperpolarizing current injection of −90 pA (Figure 1C). Sevoflurane significantly reduced the input resistance of recorded thalamocortical neurons in a dose-dependent manner starting at a concentration of 0.33 mM [control: 227.6 (181.3–276.7) MΩ, *n* = 45; 0.08 mM: −15.8 (−20.8 to +1.0) MΩ, *n* = 10, *p* > 0.999; 0.33 mM: −43.8 (−62.1 to −38.0) MΩ, *n* = 9, *p* = 0.016; 0.45 mM: −50.9 (−88.3 to −37.2) MΩ, *n* = 12, *p* = 0.003; 0.69 mM: −137.5 (−205.4 to −89.5) MΩ, *n* = 9, *p* < 0.001; 1.47 mM: −119.5 (−175.3 to −112.6) MΩ, *n* = 5, *p* < 0.001, Figure 1C]. The effect of sevoflurane on the input resistance was concentration-dependent (*r*^2^ = 0.5834, *F* = 77.02, DF_n_, DF_d_ = 1.000, 55.00, *p* < 0.001). Using non-linear regression analysis, we calculated an IC_50_ of 0.53 (95% CI: 0.31–0.89) mM sevoflurane [Hill coefficient: −2.2 (95% CI: −4.21 to 0.11), *R*^2^ = 0.6415, Figure 1C]. In a set of additional experiments, we coapplied 0.69 mM sevoflurane and the GABA_A_ receptor antagonist bicuculline (0.01 mM). By the addition of bicuculline, the sevoflurane-induced reduction of the input resistance was blocked. However, we did not observe an increase in the input resistance under these conditions (data not shown). Furthermore, we evaluated the effect of coapplying ZD7288 (0.04 mM) and 0.69 mM sevoflurane on the input resistance as well. In four out of seven cells, the input resistance increased under these conditions, with a median difference of +160.8 (−36.8 to +217.0) MΩ. However, this effect was statistically not significant compared to baseline conditions [control: 276.7 (200.3–348.6) MΩ; 0.69 mM sevoflurane + 0.04 mM ZD7288: 434.7 (163.5–600.3) MΩ, *n* = 7, *p* = 0.219, Wilcoxon matched-pairs signed-rank test]. A comparison with the effect of 0.69 mM sevoflurane on the input resistance alone [control: 297.6 (222.2–373.4) MΩ; 0.69 mM sevoflurane: 160.6 (128.5–182.0) MΩ, *n* = 9, *p* = 0.004, Wilcoxon matched-pairs signed-rank test] is depicted in Figure 1D.

In contrast to passive membrane properties, sevoflurane-induced effects on active firing patterns were less pronounced. Sevoflurane generally caused reduced neuronal excitability. Under control conditions, action potential firing of thalamocortical neurons occurred at −38.9 mV (−40.1 to −36.0 mV, *n* = 45). At 0.33 mM, sevoflurane led to a shift of the action potential threshold towards more depolarized values with comparable results over the whole range of applied concentrations [0.08 mM: +0.6 (0.0 to +1.3) mV, *n* = 10, *p* = 0.592; 0.33 mM: +2.2 (+1.5 to +2.5) mV, *n* = 9, *p* = 0.001; 0.45 mM: +1.0 (+0.3 to +2.0) mV, *n* = 12, *p* = 0.022; 0.69 mM: +1.1 (+0.4 to +3.2) mV, *n* = 9, *p* = 0.010; 1.47 mM: +3.0 (+1.1 to +4.3) mV, *n* = 5, *p* = 0.006, Figure 1E]. As depicted in Figure 1E, linear regression analysis revealed that the effect of sevoflurane on the action potential threshold was not dose-dependent (*r*^2^ = 0.0769, *F* = 3.667, DF_n_, DF_d_ = 1.000, 44.00, *p* = 0.062).

To determine the effect on tonic action potential firing, we measured the median action potential frequency following a current injection of +360 pA. Compared to results under control conditions [39.2 (33.8–43.8) Hz, *n* = 45], we observed a significant decrease of action potential frequency only at higher concentrations of 0.69 mM and 1.47 mM sevoflurane [0.08 mM: +1.3 (−0.2 to +5.2) Hz, *n* = 10, *p* > 0.999; 0.33 mM: −1.7 (−7.1 to +0.4) Hz, *n* = 9, *p* = 0.486; 0.45 mM: −3.3 (−8.8 to −0.4) Hz, *n* = 12, *p* = 0.111; 0.69 mM: −6.7 (−7.9 to −4.2) Hz, *n* = 9, *p* = 0.007; 1.47 mM: −11.5 (−24.6 to −9.3) Hz, *n* = 5, *p* < 0.001, Figure 1F]. The effect of sevoflurane on tonic action potential firing was concentration-dependent (*r*^2^ = 0.4700, *F* = 39.01, DF_n_, DF_d_ = 1.000, 44.00, *p* < 0.001, Figure 1F). To determine whether the significant reduction of action potential frequency observed in the presence of 0.69 mM sevoflurane was primarily a consequence of the reduced input resistance, we further compared the action potential frequency at 0.69 mM sevoflurane with the corresponding action potential frequency under control conditions at the current injection required to obtain approximately equal membrane potentials. As was to be expected due to the decreasing input resistance, significantly less depolarizing current injection was required under control conditions to attain comparable membrane potentials [control: +320 (260–350) pA vs. 0.69 mM sevoflurane: +360 pA, *n* = 9, *p* = 0.008]. However, the reduction in action potential frequency in the presence of sevoflurane was still statistically significant [control: 31.7 (25.8–40.0) Hz vs. 0.69 mM sevoflurane: 26.7 (21.7–34.2) Hz, *n* = 9, *p* = 0.008].

### Effects of Sevoflurane on I_h_ Current Amplitude, Voltage-Dependent Gating of HCN Channels and Functional I_h_ Activation Kinetics

Since active and passive membrane properties are regulated by an interplay of different ionic conductances, with the HCN-mediated I_h_ playing a central role (Zobeiri et al., 2017), HCN channel impairment appeared therefore as a plausible mechanism apt for further investigation.

**Figure 2:**
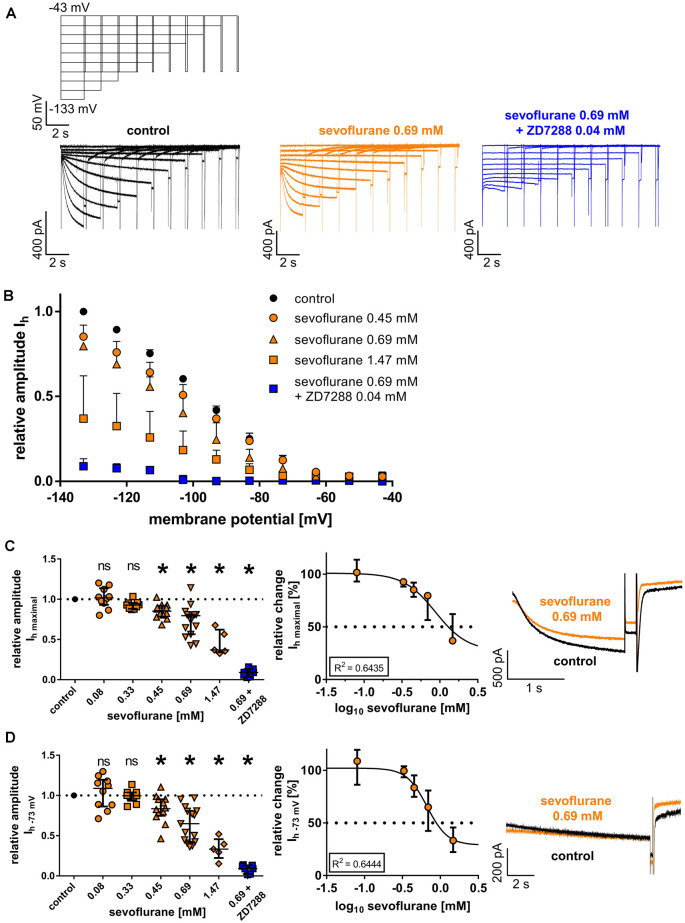
**(A)** Representative current traces from thalamocortical relay neurons under control conditions with the corresponding voltage protocol, after application of 0.69 mM sevoflurane, and after applying both 0.69 mM sevoflurane and 0.04 mM ZD7288. **(B)** Beginning from a concentration of 0.45 mM, sevoflurane significantly reduced HCN-mediated I_h_ current amplitude over a broad range of membrane potentials. **(C)** The maximum I_h_ current amplitude (I_h maximal_) is attained when the cell is hyperpolarized to −133 mV. The reduction of I_h maximal_ was dose-dependent. Corresponding current traces of I_h__maximal_ under control conditions and in presence of 0.69 mM sevoflurane are depicted on the right. ns, not significant. *p < 0.05. **(D)** The reduction of I_h_ current amplitude in the presence of sevoflurane was also observable at membrane potentials closer to a neuron’s physiological state. Again, starting from a concentration of 0.45 mM, sevoflurane inhibited I_h_ current amplitude at −73 mV and this effect was concentration-dependent. Presented on the right, representative current traces of I_h_ at −73 mV under control conditions and in the presence of 0.69 mM.

Following a series of hyperpolarizing voltage steps, all recorded thalamocortical relay neurons from the VB thalamus demonstrated pronounced native I_h_ currents with characteristic time and voltage-dependent activation (Figure 2A). As presented in Figure 2B, the reduction of I_h_ current amplitude in the presence of sevoflurane extended over a broad range of voltage steps from −73 mV to −133 mV. Upon closer examination, sevoflurane caused a significant reduction of the maximal I_h_ current amplitude at −133 mV, starting at a concentration of 0.45 mM [values relative to control: 0.08 mM: 1.02 (0.93–1.14), *n* = 10, *p* > 0.999; 0.33 mM: 0.93 (0.88–0.96), *n* = 9, *p* = 0.516; 0.45 mM: 0.85 (0.79–0.92), *n* = 12, *p* = 0.011; 0.69 mM: 0.80 (0.57–0.81), *n* = 15, *p* < 0.001; 1.47 mM: 0.37 (0.34–0.62), *n* = 5, *p* < 0.001, Figure 2C]. This observed reduction was concentration-dependent with a calculated IC_50_ of 0.88 (95% CI: 0.40–2.2) mM sevoflurane [Hill coefficient: −2.10 (95% CI: −3.91 to −0.20), *R*^2^ = 0.6435, Figure 2C]. In a set of additional experiments, we coapplied 0.69 mM sevoflurane and the selective HCN channel antagonist ZD7288 (0.04 mM), which significantly and almost completely decreased the maximal I_h_ current amplitude [value relative to control: 0.09 (0.04–0.13), *n* = 7, *p* = 0.016, Wilcoxon matched-pairs signed-rank test].

When measured at −73 mV, reflecting a membrane potential closer to a physiologically relevant state, the reduction of I_h_ was still significant, with differences reaching significance again at a concentration of 0.45 mM sevoflurane [values relative to control: 0.08 mM: 1.09 (0.86–1.20), *n* = 10, *p* > 0.999; 0.33 mM: 1.00 (0.93–1.04), *n* = 9, *p* > 0.999; 0.45 mM: 0.84 (0.75–0.95), *n* = 12, *p* = 0.023; 0.69 mM: 0.65 (0.43–0.81), *n* = 15, *p* < 0.001; 1.47 mM: 0.33 (0.22–0.46), *n* = 5, *p* < 0.001, Figure 2D]. The observed reduction of I_h_ at −73 mV was also dose-dependent, yielding a calculated IC_50_ of 0.67 (95% CI: 0.51–0.87) mM sevoflurane [Hill coefficient: −3.22 (95% CI: −5.36 to −1.07), *R*^2^ = 0.6444, Figure 2D]. Again, the addition of ZD7288 (0.04 mM) to 0.69 mM sevoflurane resulted in an almost complete suspension of I_h_ at this membrane potential [value relative to control: 0.09 (0.03–0.13), *n* = 7, *p* = 0.016, Wilcoxon matched-pairs signed-rank test].

**Figure 3:**
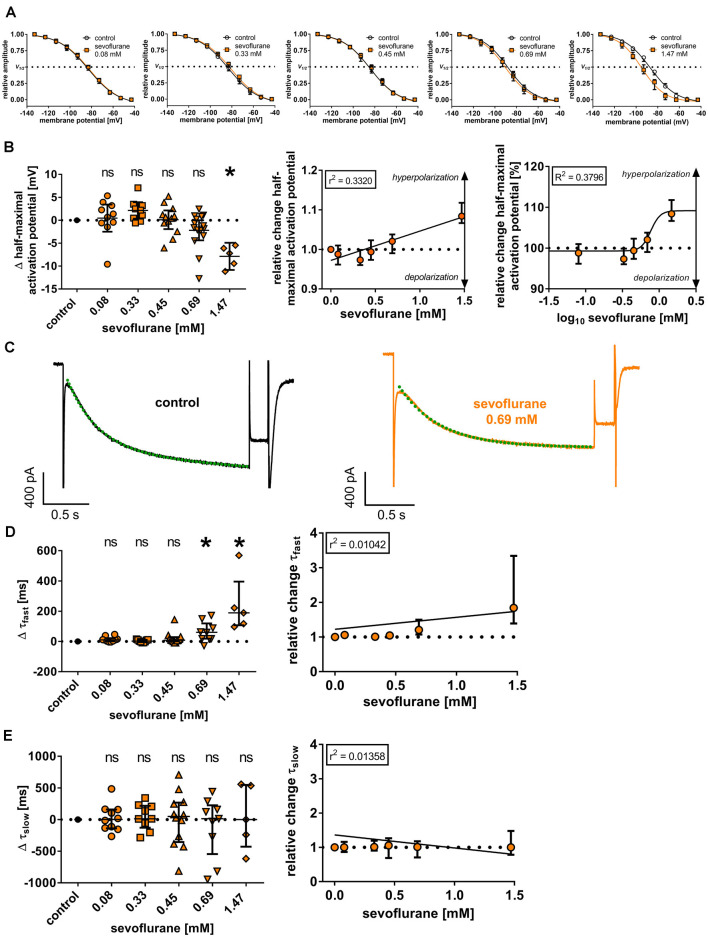
**(A)** Normalized tail currents (I_tail_) were fitted to a curve using a Boltzmann function to analyze voltage-dependent channel gating under control conditions and in the presence of increasing concentrations of sevoflurane. V_1/2_: half-maximal activation voltage. **(B)** In a concentration range of 0.08 mM to 0.69 mM, we did not observe a change of the half-maximal activation voltage [control: −85.6 (−88.2 to −83.2) mV, *n* = 51], only 1.47 mM sevoflurane significantly shifted the half-maximal activation voltage towards hyperpolarization. Using linear regression analysis and non-linear regression analysis, the shift towards hyperpolarization was concentration-dependent. Δ: absolute change compared to control. ns, not significant. *p < 0.05. **(C)** Representative I_h_ current traces at −133 mV under control conditions and after applying 0.69 mM sevoflurane. Activation kinetics of I_h_ were acquired by fitting a biexponential function to the I_h_ current at −133 mV, resulting in a fast (τ_fast_) and a slow (τ_slow_) time constant of time-dependent activation. The biexponential curve fitting is visualized with green dotted lines. **(D)** Higher concentrations of 0.69 mM and 1.47 mM resulted in a significant prolongation of τ_fast_ [control: 218 (183–256) ms, *n* = 45], whereas no statistically significant differences concerning τ_slow_ [control: 1,091 (979–1,373) ms, *n* = 45] were observed **(E)**. In both cases, changes were not concentration-dependent.

The reduction of I_h_ in the presence of sevoflurane might be attributable to a reduction in open channel probability by changing the voltage dependency of HCN channel activation. In the case of thalamocortical relay neurons, this mechanism of action was shown for several anesthetic agents (Ying et al., 2006; Budde et al., 2008; Mattusch et al., 2015) and it was postulated that sevoflurane might have a similar effect (Budde et al., 2008). Under control conditions, the half-maximal activation potential was −85.6 mV (−88.2 to −83.2 mV, *n* = 51), showing good accordance with previously published values for thalamocortical relay neurons (Budde et al., 2008; Datunashvili et al., 2018; Zobeiri et al., 2019). Interestingly, sevoflurane application in the extracellular solution did not significantly change the half-maximal activation potential of HCN channels in the concentration range of 0.08 mM to 0.69 mM, which reflects the concentrations with the highest clinical relevance [0.08 mM: +1.0 (−0.8 to +3.4) mV, *n* = 10, *p* > 0.999; 0.33 mM: +2.2 (+0.1 to +3.3) mV, *n* = 9, *p* = 0.342; 0.45 mM: +0.5 (−2.0 to +2.2) mV, *n* = 12, *p* > 0.999; 0.69 mM: −1.6 (−3.4 to +0.9) mV, *n* = 15, *p* = 0.966, Figures 3A,B]. Only a high concentration of 1.47 mM induced a significant shift of the half-maximal activation potential towards hyperpolarization [−7.2 (−10.3 to −5.8) mV, *n* = 5, *p* = 0.020], suggesting a reduced probability of channel opening (Figures 3A,B). However, linear regression revealed that the shift of the half-maximal activation potential towards hyperpolarization was still concentration-dependent (*r*^2^ = 0.3320, *F* = 24.85, DF_n_, DF_d_ = 1.000, 50.00, *p* < 0.001, Figure 3B). Within this range, we calculated an EC_50_ of 0.77 (95% CI: 0.50–1.17) mM for sevoflurane’s effect on the half-maximal activation potential [Hill coefficient: 6.76 (95% CI: −16.19 to +29.72), *R*^2^ = 0.3796, Figure 3B]. As a limiting factor, it should be noted that we observed substantial leak currents at 1.47 mM sevoflurane despite the addition of 0.15 mM barium to the extracellular solution, which required adjustments to the conventional protocol to determine the half-maximal activation potential by normalizing I_tail_ currents. This problem has already been encountered and described elsewhere, in which case even higher concentrations of barium had been used (Budde et al., 2008).

We further examined whether sevoflurane has an impact on the time course of I_h_ activation, which in turn is substantially shaped by the length and magnitude of the voltage step analyzed. The activation constants in these sets of experiments averaged 218 ms (183–256 ms; *n* = 45) for τ_fast_ and 1,091 ms (979–1,373 ms; *n* = 45) for τ_slow_ under baseline conditions. Sevoflurane, at least at higher concentrations, slowed the fast component of I_h_ activation, with significant differences observable at 0.69 mM [0.08 mM: +10 (+1 to +22) ms, *n* = 10, *p* = 0.436; 0.33 mM: +2 (−5 to +11) ms, *n* = 9, *p* > 0.999; 0.45 mM: +8 (+1 to +28) ms, *n* = 12, *p* > 0.338; 0.69 mM: +60 (+18 to +119) ms, *n* = 9, *p* = 0.003; 1.47 mM: +189 (+107 to +395) ms, *n* = 5, *p* < 0.001, Figure 3D]. However, applying linear regression analysis, this effect was not concentration-dependent (*r*^2^ = 0.0104, *F* = 0.5790, DF_n_, DF_d_ = 1.000, 55.00, *p* = 0.450, Figure 3D). By contrast, τ_slow_ was not affected by sevoflurane application [0.08 mM: +2 (−139 to +159) ms, *n* = 10, *p* = 0.997; 0.33 mM: +12 (−129 to +217) ms, *n* = 9, *p* = 0.997; 0.45 mM: +50 (−355 to +268) ms, *n* = 12, *p* = 0.997; 0.69 mM: +14 (−546 to +225) ms, *n* = 9, *p* = 0.940; 1.47 mM: +0 (−429 to +546) ms, *n* = 5, *p* < 0.001, Figure 3E] and there was no apparent concentration dependence (*r*^2^ = 0.0136, *F* = 0.7570, DF_n_, DF_d_ = 1.000, 50.00, *p* = 0.388, Figure 3E).

### Effects of Sevoflurane on I_h_ Dependent Voltage Sag and Burst Firing During Rebound Depolarization

**Figure 4:**
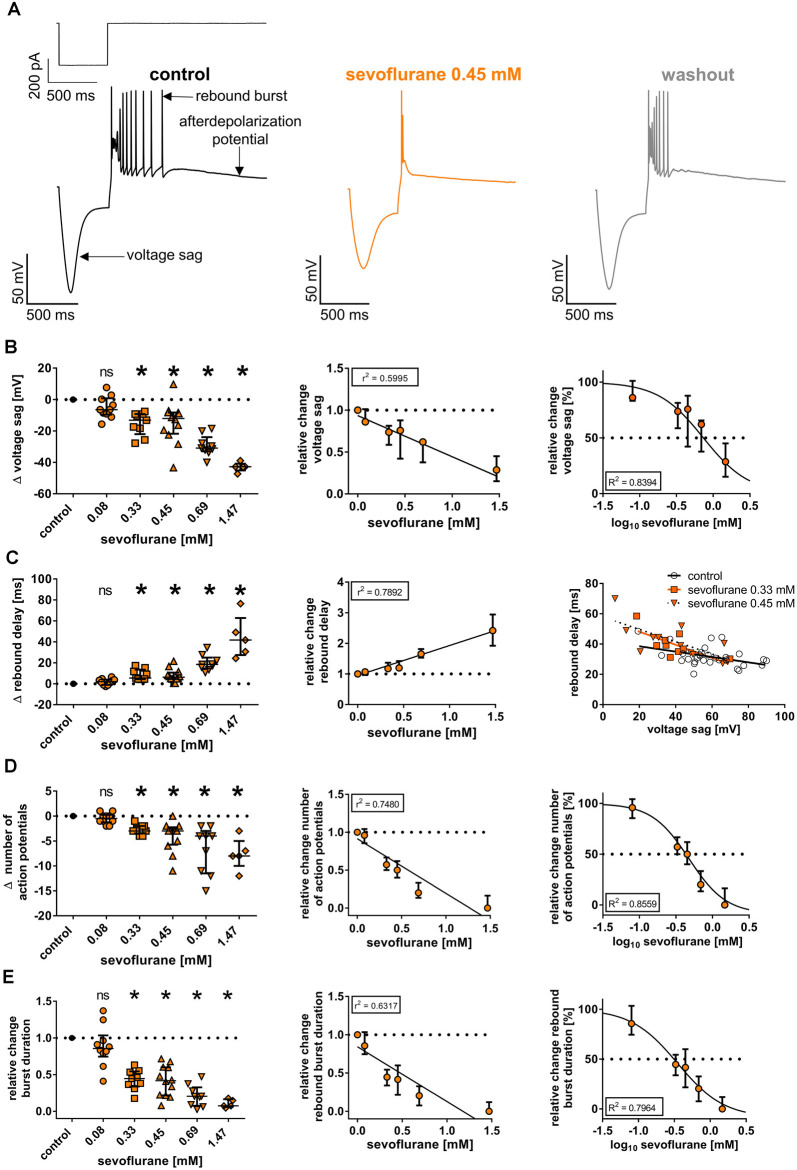
**(A)** Upon a hyperpolarizing current injection (−350 pA, 500 ms), thalamocortical relay neurons demonstrated a prominent, inwardly directed rectification of the membrane potential (voltage sag), followed by low-threshold calcium spike rebound burst firing (rebound burst) and an afterdepolarization potential. Exemplar membrane voltage traces under control conditions (with current protocol shown above), in the presence of 0.45 mM sevoflurane, and after a 20-min washout. **(B)** Beginning at a concentration of 0.33 mM, sevoflurane significantly impaired the voltage sag [control: 57.9 (49.9–73.3) mV, *n* = 45]. The reduction occurred in a concentration-dependent manner, using linear and non-linear regression analysis. Δ: absolute change compared to control. ns, not significant. *p < 0.05. **(C)** Conversely, sevoflurane significantly prolonged the rebound burst delay [control: 30.0 (26.9–33.6) ms, *n* = 45] starting from a concentration of 0.33 mM in a dose-dependent fashion. On the right, voltage sag amplitudes are plotted as a function of rebound delay under control conditions and in the presence of 0.33 mM and 0.45 mM sevoflurane with corresponding regression lines. Following previous findings, correlation analysis revealed an inverse correlation between voltage sag amplitude and rebound burst delay, which was intensified with increasing sevoflurane concentrations. **(D)** Further, sevoflurane significantly decreased the number of action potentials seen during rebound burst spiking [control: 7.0 (5.0–8.5) mV, *n* = 45] beginning at a concentration of 0.33 mM and the reduction was mediated in a concentration-dependent manner. **(E)** Lastly, sevoflurane significantly abbreviated burst duration [control: 74.0 (57.0–122.3) ms, *n* = 45] starting at a concentration of 0.33 mM in a dose-dependent fashion.

To investigate if the observed effects of sevoflurane on HCN channel function in thalamocortical relay neurons also translate to parameters of neuronal excitability and thalamic burst firing patterns associated with HCN channels, we conducted additional whole-cell current-clamp recordings and analyzed the effects of sevoflurane on the voltage sag and rebound burst delay as well as the duration of subsequent rebound bursts and the number of action potentials observed during burst activation at different concentrations. Following an intracellular hyperpolarizing current injection of −350 pA and in the presence of 0.15 mM barium, thalamocortical relay neurons of the VB complex displayed a prominent voltage sag of 57.9 mV (49.9–73.3 mV, *n* = 45, Figure 4A). Sevoflurane sharply decreased the voltage sag amplitude starting at 0.33 mM sevoflurane [0.08 mM: −6.4 (−9.6 to +0.8) mV, *n* = 10, *p* > 0.999; 0.33 mM: −13.2 (−22.0 to −9.2) mV, *n* = 9, *p* = 0.011; 0.45 mM: −12.0 (−21.7 to −8.3) mV, *n* = 12, *p* = 0.013; 0.69 mM: −30.9 (−32.9 to −23.9) mV, *n* = 9, *p* < 0.001; 1.47 mM: −42.7 (−45.1 to −40.8) mV, *n* = 5, *p* < 0.001, Figure 4B]. This effect was concentration-dependent (*r*^2^ = 0.5995, *F* = 82.3, DF_n_, DF_d_ = 1.000, 55.00, *p* ≤ 0.001, Figure 4B). Non-linear curve fitting of sevoflurane’s effect on the voltage sag yielded a calculated IC_50_ of 0.74 mM [95% CI: 0.62–0.87 mM; Hill coefficient: −1.40 (95% CI: −1.8 to −1.0), *R*^2^ = 0.8394, Figure 4B].

As expected and presupposing an inverse correlation with decreasing voltage sags, application of sevoflurane resulted in a prolongation of the rebound delay. Again, significant changes were observed beginning at a concentration of 0.33 mM [control: 30.0 (26.9–33.6) ms, *n* = 45; 0.08 mM: +2.0 (−1.1 to +4.0) ms, *n* = 10, *p* > 0.999; 0.33 mM: +5.5 (+4.4 to +13.5) ms, *n* = 9, *p* = 0.007; 0.45 mM: +6.3 (+4.6 to +10.6) ms, *n* = 12, *p* = 0.004; 0.69 mM: +18.5 (+14.6 to +25.0) ms, *n* = 9, *p* < 0.001; 1.47 mM: +41.8 (+27.4 to +62.8) ms, *n* = 5, *p* < 0.001, Figure 4C]. The prolongation of the rebound delay was concentration-dependent (*r*^2^ = 0.7892, *F* = 205.9, DF_n_, DF_d_ = 1.000, 55.00, *p* ≤ 0.001, Figure 4C). This is reflected by correlation analysis with the determination of corresponding Pearson correlation coefficients (*r*), which we performed exemplarily for 0.33 mM and 0.45 mM sevoflurane. The inverse correlation between voltage sag and rebound delay was increased, when exposed to sevoflurane [*r* at control: −0.445 (95% CI: −0.694 to −0.100), *p* = 0.014; *r* at 0.33 mM: −0.694 (95% CI: −0.930 to −0.055), *p* = 0.038; *r* at 0.45 mM: −0.722 (95% CI: −0.916 to −0.253), *p* = 0.008] with a steepening of the linear relationship’s slope (voltage sag depicted as a function of rebound delay, Figure 4C).

Further, sevoflurane decreased the number of action potentials during rebound spike activation. Under control conditions, rebound bursts displayed a median of 7.0 (5.0–8.5) action potentials (*n* = 45). Beginning at a concentration of 0.33 mM, the number of action potentials decreased significantly [0.08 mM: by −0.5 (−1.3 to +0.3) action potentials, *n* = 10, *p* > 0.999; 0.33 mM: by −3.0 (−3.5 to −2.0) action potentials, *n* = 9, *p* = 0.010; 0.45 mM: by −3.0 (−5.8 to −2.3) action potentials, *n* = 12, *p* < 0.001; 0.69 mM: by −4.0 (−11.5 to −3.0) action potentials, *n* = 9, *p* < 0.001; 1.47 mM: by −8.0 (−10.0 to −5.0) action potentials, *n* = 5, *p* < 0.001, Figure 4D]. The reduction of action potentials was concentration-dependent (*r*^2^ = 0.7480, *F* = 163.3, DF_n_, DF_d_ = 1.000, 55.000, *p* < 0.001, Figure 4D). Non-linear regression analysis rendered an IC_50_ of 0.48 mM [95% CI: 0.32–0.71 mM; Hill coefficient: −1.71 (95% CI: −2.7 to −0.7), *R*^2^ = 0.8559, Figure 4D].

Correspondingly, the average burst duration of 74.0 ms (57.0–122.3 ms, *n* = 45) under baseline conditions became significantly shorter, beginning at a concentration of 0.33 mM [values relative to control: 0.08 mM: 0.86 (0.75–1.04), *n* = 10, *p* > 0.999; 0.33 mM: 0.45 (0.34–0.54), *n* = 9, *p* = 0.003; 0.45 mM: 0.42 (0.22–0.60), *n* = 12, *p* < 0.001; 0.69 mM: 0.20 (0.08–0.33), *n* = 9, *p* < 0.001; 1.47 mM: 0.07 (0.06–0.16) ms, *n* = 5, *p* < 0.001, Figure 4E]. Again, this effect was dose dependent (*r*^2^ = 0.6317, *F* = 94.31, DF_n_, DF_d_ = 1.000, 55.000, *p* < 0.001, Figure 4E). Non-linear regression analysis yielded an IC_50_ of 0.30 mM [95% CI: 0.18–0.50 mM; Hill coefficient: −1.5 (95% CI: −2.3 to −0.6), *R*^2^ = 0.7964, Figure 4E].

Absolute values from pre- and post-sevoflurane application for each parameter analyzed are presented in Supplementary Table 1 through Supplementary Table 12, itemized according to the different concentration groups.

## Discussion

In this study, we demonstrate that the volatile anesthetic sevoflurane suppresses the I_h_ conductance mediated by native HCN channels and substantially reduces the excitability of thalamocortical relay neurons. Many of these effects show a distinct dose-dependence and potentially contribute to the anesthetic properties of sevoflurane.

A major concern in the research about the evaluation of volatile anesthetics’ molecular targets constitutes the application of clinically relevant concentrations (Eger et al., 2001). Volatile anesthetics are usually defined by their minimum alveolar concentration (MAC; Eger et al., 1965). However, the aqueous concentration resulting from the administration of sevoflurane in the gas phase is temperature-dependent and decreases with increasing temperatures (Franks and Lieb, 1996). As we performed electrophysiological *in vitro* experiments at room temperature (20–24°C), using a 1.0 MAC could underestimate actual aqueous concentrations. Therefore, we performed headspace gas chromatographic measurements and our results (0.24 mM per vol.% sevoflurane) are well within the range of previously published data, which vary between 0.08 mM per vol.% (Nishikawa et al., 2011) and 0.21 mM per vol.% (Haseneder et al., 2009) sevoflurane. For *in vitro* experiments conducted at room temperature, presuming an EC_50_ of 0.33 mM sevoflurane was recommended (Garcia et al., 2010). In an earlier study (Haseneder et al., 2009), we determined that a MAC of 1.0 for sevoflurane for mice corresponds to an aqueous concentration of 0.38 mM when appropriate partition coefficients and temperature corrections (Franks and Lieb, 1993, 1996) are employed. Therefore, we consider the concentrations of sevoflurane used in this study (between 0.08 and 0.69 mM) to represent a clinically relevant range, whereas the high concentration of 1.47 mM was deliberately chosen as basis for the generation of concentration-response curves.

The VB complex and adjacent thalamic nuclei comprise primarily first-order relays conveying peripheral sensory input to the cortex (Sherman and Guillery, 1996). Even though the cerebral cortex might maintain a certain level of responsiveness under anesthesia (Hudetz et al., 2009; Raz et al., 2014), the effects of anesthetic agents on thalamocortical relay neurons could still alter the spectrum of information transfer (Mashour and Hudetz, 2017) or impair corticocortical communication (Guillery and Sherman, 2002). In the end, a functional dissociation of cortical from subcortical components may prove to be a difficult task, as cortical and subcortical networks are highly interconnected and effects of anesthetic agents are manifold (Mashour and Hudetz, 2017). However, whether a region of interest displays sensitivity towards an anesthetic within a clinically relevant concentration range or not is crucial to understand its relative importance (Voss et al., 2019). Therefore, it is questionable whether the effects of sevoflurane on thalamocortical relay neurons described in this study, which exclusively occur at the high to very high concentration range (>0.69 mM sevoflurane), play a decisive role in mediating relevant clinical endpoints, like loss of consciousness.

Previously, we showed that sevoflurane dose-dependently reduces cortical depolarization in all cortical layers following stimulation of the VB thalamus and prolongs thalamocortical signal propagation *in vitro* (Kratzer et al., 2017). Sevoflurane-induced changes in thalamic activity may therefore interfere with normal information processing. The thalamus presents a crucial target site of anesthetic action (Franks, 2008; Mashour and Hudetz, 2017). Most anesthetic agents suppress thalamic metabolism and blood flow and impair thalamocortical connectivity (Alkire et al., 2000; Ranft et al., 2016). Additionally, various models of dynamic systems show that changes in thalamic activity are sufficient to induce cortical and thalamocortical oscillations observed under general anesthesia (Ching and Brown, 2014). These electroencephalographic patterns associated with anesthesia include synchronous, slow oscillations of the δ-frequency range (1–4 Hz) which are actively shaped by thalamic networks (Neske, 2016). Cyclic activation of HCN channel-mediated I_h_ is regarded as a molecular prerequisite for thalamocortical relay neurons to generate and synchronize their burst firing activity, acting as a pacemaker current for δ-oscillations (McCormick and Pape, 1990; Destexhe and Sejnowski, 2003). A reduction of I_h_ is associated with a deceleration of intrathalamic oscillation frequencies as well as an attenuation of their regularity (Yue and Huguenard, 2001; Zobeiri et al., 2019). This effect was reproduced pharmacologically with the anesthetic propofol (Ying et al., 2006), underscoring the importance of HCN channel-mediated I_h_ for thalamic activity.

Sevoflurane acts on a broad range of molecular targets: besides enhancing inhibition *via* GABA_A_ receptors (Garcia et al., 2010), it also affects glutamate and glycine receptors (Rudolph and Antkowiak, 2004). In contrast, our understanding of its interaction with HCN channels is still emerging. The HCN gene family comprises four subunits (HCN1–HCN4) which can assemble as homo- and heterotetramers (Altomare et al., 2003). There are profound differences between HCN channel isoforms concerning their functional properties and activation kinetics as well as their physiological and pharmacological regulation (Riegelhaupt et al., 2018). Additionally, regional brain compartments show distinct expression patterns of HCN channel isoforms (Notomi and Shigemoto, 2004). Concerning thalamocortical relay neurons from the VB complex, the evidence is mounting that HCN2 and HCN4 are paramount. These two isoforms not only show the highest levels of transcript and strong immunoreactivity but also appear to be functionally most relevant (Ludwig et al., 2003; Notomi and Shigemoto, 2004; Meuth et al., 2006; Zobeiri et al., 2019). This indicates that I_h_ in thalamocortical relay neurons is primarily mediated by HCN2 and HCN4. Furthermore, thalamocortical relay neurons of the VB complex not only demonstrate strong expression rates of HCN2 and HCN4 on dendritic spines and shafts but also on the soma (Abbas et al., 2006). This constitutes a marked difference to other neurons, for example, inhibitory interneurons of the reticular thalamic nucleus (Abbas et al., 2006) or hippocampal CA1 pyramidal cells (Lörincz et al., 2002), where I_h_ is predominately shaped by HCN channels located distant from the soma.

Furthermore, as data from homomeric HCN channels expressed in HEK293 suggests, volatile anesthetics may affect HCN channels in a subunit-specific manner. For halothane and isoflurane, the hyperpolarizing shift in the half-maximal activation potential is principally conveyed by HCN1, whereas cells expressing HCN2 showed a reduction of I_h_ current amplitude with no significant effect on the voltage dependency of activation. Further, halothane leads to a slowing of current activation in the case of HCN2 with an opposite effect for HCN1 (Chen et al., 2005). Comparable to our results, a marked divergence between a reduction of I_h_ current amplitude and a rather modest shift in the voltage dependence of I_h_ activation was also observed in hypoglossal motoneurons in the presence of halothane (Sirois et al., 1998) and in cholinergic interneurons of the striatum in the presence of sevoflurane (Sugasawa et al., 2018). In the latter case, these neurons mainly express HCN2 and HCN4 subunits as well (Santoro et al., 2000). Consistent with our findings, sevoflurane dose-dependently reduced maximal I_h_ current amplitude, whereas 4.0 vol.% sevoflurane (recordings performed at 30°C) only induced a modest shift in the half-activation potential towards hyperpolarization (−4.1 mV). The authors of this study therefore hypothesized that sevoflurane acts as an HCN channel modulator rather than a channel blocker (Sugasawa et al., 2018). Correspondingly, we found that coapplication of the selective HCN channel blocker ZD7288 (0.04 mM) with sevoflurane resulted in a markedly stronger reduction of I_h_ maximal current amplitude as well as an additional hyperpolarization of the resting membrane potential. When using propofol—surmized to have an occluding effect on HCN channels of thalamocortical relay neurons—the addition of ZD7288 did in contrast not result in an additional reduction of I_h_ (Ying et al., 2006).

As mentioned above, we observed a slowing of τ_fast_ of HCN activation kinetics, but only at a concentration of 0.69 mM sevoflurane which may mitigate its clinical relevance (Figure 3D). There were no significant changes concerning τ_slow_ (Figure 3E). However, the validity of our results about τ_slow_ could be thwarted by the fact that the applied voltage-step duration of 2 s was too short to yield currents approaching adequate equilibrium (Riegelhaupt et al., 2018). Depending on the subunit composition, HCN channels demonstrate differences regarding their activation kinetics (Kaupp and Seifert, 2001). Compared to HCN1, HCN2–HCN4 require substantially longer voltage steps for stable-state values (Seifert et al., 1999). As neurons in acute brain slices do not easily endure membrane hyperpolarization of −110 mV or more (Riegelhaupt et al., 2018), we used a protocol with variable voltage-step durations to ensure stable recordings, but this might constitute a caveat to the slow component of HCN channel activation (τ_slow_).

Regardless of the exact mode of action, sevoflurane substantially attenuated I_h_-dependent membrane properties of thalamocortical relay neurons in a dose-dependent manner, as it reduced the voltage sag and number of action potentials during subsequent rebound bursts, shortened the duration of rebound bursts as well as prolonged the latency until rebound burst firing activity (Figure 4). A prominent voltage sag upon hyperpolarizing current injections is a hallmark of thalamocortical relay neurons and induces low-threshold calcium spikes (*via* T-type calcium channels) with a burst of action potentials once hyperpolarization ends (Ying et al., 2006). The voltage sag is dependent on HCN channels and in the case of thalamocortical relay neurons most likely on HCN2 and HCN4, as corresponding gene deletion resulted in a pronounced reduction of the voltage sag (Ludwig et al., 2003; Zobeiri et al., 2019). This closely resembles the findings of other studies, which demonstrated inhibition of HCN channel function in thalamocortical relay neurons with propofol and xenon (Ying et al., 2006; Mattusch et al., 2015), suggesting, in turn, an inhibitory effect of sevoflurane. However, the effects of sevoflurane on the rebound burst firing are quite likely also shaped by the sevoflurane-induced decrease of the input resistance and potential inhibition of T-type calcium channels (Ries and Puil, 1999; Budde et al., 2008).

Another observation requires discussion, as HCN channel inhibition seems to increase neuronal input resistance, promoting action potential generation at excitatory synapses (Biel et al., 2009). However, many anesthetic agents reduce the input resistance and sevoflurane led to a dose-dependent decrease as well (Figure 1C). This strong, dose-dependent decrease of the input resistance is also likely to contribute in part to the other changes of active and passive membrane properties presented in this study. In the case of propofol, this effect is mediated by GABA_A_ receptors (Ying and Goldstein, 2005; Ying et al., 2006). Correspondingly, we could previously show that xenon results in an increase in the input resistance (Mattusch et al., 2015). While there are no substantial effects of xenon on GABA_A_ receptors (Kubota et al., 2020), it inhibits HCN channel-mediated I_h_ by shifting the half-maximal activation potential towards hyperpolarization (Mattusch et al., 2015). As sevoflurane is a known GABA_A_ receptor agonist (Brohan and Goudra, 2017), this might sufficiently explain the dose-dependent reduction of the neuronal input resistance. Accordingly, we found that the decrease of the input resistance in presence of 0.69 mM sevoflurane was blocked when adding GABA_A_ receptor antagonist bicuculline to the bath solution (with the caveat that the input resistance was not increased either—a similar observation has been made when coapplying propofol and bicuculline to thalamocortical relay neurons, Ying et al., 2006). The HCN channel blocker ZD7288, which is known to strongly increase neuronal input resistance, caused an increase of the median input resistance in the presence of 0.69 mM sevoflurane. However, this observed effect was neither consistent nor statistically significant. Again, this resembles previous findings, where coapplication of propofol and ZD7288 did not have a statistically significant impact on the input resistance of thalamocortical relay neurons (Ying et al., 2006). Further, inhibition of I_h_ and I_h_-mediated voltage sag appear to be neither affected by the coapplication of the GABA_A_ receptor antagonist bicuculline nor of GABA, suggesting a mechanism independent of GABAergic signaling (Frere and Luthi, 2004; van Welie et al., 2004; Ying et al., 2006). In summary, the results of this study are in agreement with existing literature and indicate an involvement of GABA_A_ receptors in mediating the reduction of the neuronal input resistance.

Concerning the resting membrane potential of thalamocortical relay neurons, inhibition of HCN channel function usually results in hyperpolarization (Meuth et al., 2006). At least at higher concentrations, sevoflurane induced a significant hyperpolarization of the resting membrane potential. This effect was further enhanced by the addition of ZD7288. However, it should be noted that the resting membrane potential of thalamocortical relay neurons varies considerably between different species and thalamic nuclei. Published values range from −55 mV to −75 mV (Zobeiri et al., 2019). For murine thalamocortical relay neurons of the ventral posteromedial nucleus, which belongs to the VB complex, a more recent study states a resting membrane potential of −55.7 mV (Liu et al., 2015). This is in good accordance with the value presented in this work [−56.9 mV (−58.7 to −55.6 mV), *n* = 45].

Naturally, the findings of our study should be interpreted in the light of its limitations. By using acutely prepared brain slices, we chose an experimental approach that allowed us to investigate the relevant effects of sevoflurane within a relatively intact neuronal network (Voss et al., 2019). As a drawback, we cannot positively exclude potentially contaminating influences on our recordings (for example sodium or low- and high-voltage-activated calcium currents, Ying et al., 2006), but we tried to limit interfering factors through experimental considerations as much as possible.

Another limitation presents the absence of an investigation of whether sevoflurane-induced changes of HCN channel function are mediated by effects on intracellular 3′-5′-cyclic adenosine monophosphate (cAMP) metabolism. As a salient feature, HCN channel gating is not only facilitated by voltage changes, but also by cyclic nucleotides (Lewis et al., 2010). Binding of cAMP to a carboxyl-terminal cyclic nucleotide-binding domain suspends tonic inhibition, induces a substantial shift of the half-maximal activation potential towards depolarization (Ludwig et al., 1998; Wainger et al., 2001; Ying et al., 2006), enhances I_h_ currents (Robinson and Siegelbaum, 2003; Frere and Luthi, 2004; He et al., 2014) as well as significantly accelerates I_h_ activation kinetics (Wang et al., 2002). Again, cAMP gating demonstrates differences concerning HCN isoforms. HCN2 and HCN4 are substantially gated by cAMP (Wang et al., 2001), whereas HCN1 shows limited and HCN3 no cAMP gating (Stieber et al., 2005; Riegelhaupt et al., 2018). Yet cAMP-independent mechanisms of I_h_ modulation exist (van Welie et al., 2004) and the inhibitory effect of propofol was characterized as such (Ying et al., 2006; Lyashchenko et al., 2007). In the case of xenon (Mattusch et al., 2015) and the volatile anesthetics isoflurane and halothane (Chen et al., 2005), stabilization of HCN channels in the closed state was cAMP-dependent. Interestingly, the predominant effect of halothane on HCN channel function in thalamocortical relay neurons correlated with intracellular cAMP concentrations. In the presence of high levels of cAMP, halothane-dependent inhibition of HCN channels resulted mainly from a hyperpolarizing shift in V_1/2_ rather than a reduction of I_h_ current amplitude (Chen et al., 2005). Conversely, as we observed a reduction of I_h_ current amplitude instead of pronounced gating changes in the presence of sevoflurane, this effect might be partially influenced by reduced levels of intracellular cAMP. The influence of sevoflurane on intracellular cAMP could be a promising objective of future inquiries, as existing data about this subject are sparse. In neurons of the dorsal hippocampus of rats, exposure to sevoflurane resulted in lower levels of cAMP compared to control conditions (Xiong et al., 2013). This reduction of cAMP might be mediated by an inhibitory effect of sevoflurane on adenylyl cyclase activity (Kuroda et al., 2004).

Another possible limitation of this study constitutes the fact that we only used female mice. Female C57Bl/6N mice between the age of P28 and P35 have usually not reached full sexual maturity or oestrus, but they might undergo puberty at this time (Whary et al., 2015). We have found no indications of sex differences concerning neuronal HCN channel expression or function in the existing literature (He et al., 2014; Riegelhaupt et al., 2018), but we cannot definitively exclude them either.

In summary, we showed that sevoflurane dose-dependently inhibits native HCN channel function in thalamocortical relay neurons of the VB complex. Our data suggest that the hyperpolarizing shift observed under sevoflurane is rather modest and it is questionable whether this constitutes a primary mode of action. Instead, sevoflurane substantially reduced the I_h_ current amplitude in a dose-dependent manner. In line with previous findings, the observed effects of sevoflurane on HCN channel function suggest a complex inhibitory modulation instead of a channel block (Sugasawa et al., 2018). This constitutes a notable difference compared to other anesthetic agents like propofol (Ying et al., 2006). Considering the crucial role of HCN channel-mediated I_h_ in regulating membrane properties of thalamocortical relay neurons and in contributing to oscillogenesis in neuronal networks (Biel et al., 2009; He et al., 2014), the described effects of sevoflurane likely constitute an important component of its hypnotic properties.

## Data Availability Statement

The raw data supporting the conclusions of this article will be made available by the authors, without undue reservation.

## Ethics Statement

The animal study was reviewed and approved by the Ethical Committee on Animal Care and Use of the Government of Bavaria (Munich, Germany).

## Author Contributions

SS performed the experiments, conducted the data analysis, and wrote the manuscript. CK assisted with experiments and provided critical feedback on manuscript. EP took part in design and interpretation as well as provided critical feedback on the manuscript. GS provided critical feedback on manuscript. MK supported analysis and provided critical feedback on manuscript. RH and SK designed the study and analysis, overviewed interpretation and discussion, and contributed to writing the manuscript. All authors contributed to the article and approved the submitted version.

## Conflict of Interest

The authors declare that the research was conducted in the absence of any commercial or financial relationships that could be construed as a potential conflict of interest.

## References

[B1] Abbas S. Y. Ying S. W. Goldstein P. A. (2006). Compartmental distribution of hyperpolarization-activated cyclic-nucleotide-gated channel 2 and hyperpolarization-activated cyclic-nucleotide-gated channel 4 in thalamic reticular and thalamocortical relay neurons. Neuroscience 141, 1811–1825. 10.1016/j.neuroscience.2006.05.034 16806719

[B2] Agmon A. Connors B. W. (1991). Thalamocortical responses of mouse somatosensory (barrel) cortex *in vitro*. Neuroscience 41, 365–379. 10.1016/0306-4522(91)90333-j 1870696

[B3] Alkire M. T. Haier R. J. Fallon J. H. (2000). Toward a unified theory of narcosis: brain imaging evidence for a thalamocortical switch as the neurophysiologic basis of anesthetic-induced unconsciousness. Conscious. Cogn. 9, 370–386. 10.1006/ccog.1999.0423 10993665

[B4] Alkire M. T. Miller J. (2005). General anesthesia and the neural correlates of consciousness. Prog. Brain Res. 150, 229–244. 10.1016/S0079-6123(05)50017-7 16186027

[B5] Altomare C. Terragni B. Brioschi C. Milanesi R. Pagliuca C. Viscomi C.. (2003). Heteromeric HCN1-HCN4 channels: a comparison with native pacemaker channels from the rabbit sinoatrial node. J. Physiol. 549, 347–359. 10.1113/jphysiol.2002.027698 12702747 PMC2342966

[B6] Baker R. Gent T. C. Yang Q. Parker S. Vyssotski A. L. Wisden W.. (2014). Altered activity in the central medial thalamus precedes changes in the neocortex during transitions into both sleep and propofol anesthesia. J. Neurosci. 34, 13326–13335. 10.1523/JNEUROSCI.1519-14.2014 25274812 PMC4180471

[B7] Biel M. Wahl-Schott C. Michalakis S. Zong X. (2009). Hyperpolarization-activated cation channels: from genes to function. Physiol. Rev. 89, 847–885. 10.1152/physrev.00029.2008 19584315

[B8] Brioni J. D. Varughese S. Ahmed R. Bein B. (2017). A clinical review of inhalation anesthesia with sevoflurane: from early research to emerging topics. J. Anesth. 31, 764–778. 10.1007/s00540-017-2375-6 28585095 PMC5640726

[B9] Brohan J. Goudra B. G. (2017). The role of GABA receptor agonists in anesthesia and sedation. CNS Drugs 31, 845–856. 10.1007/s40263-017-0463-7 29039138

[B10] Budde T. Coulon P. Pawlowski M. Meuth P. Kanyshkova T. Japes A.. (2008). Reciprocal modulation of I (h) and I (TASK) in thalamocortical relay neurons by halothane. Pflugers Arch. 456, 1061–1073. 10.1007/s00424-008-0482-9 18478257

[B11] Chen X. Sirois J. E. Lei Q. Talley E. M. Lynch C. III Bayliss D. A. (2005). HCN subunit-specific and cAMP-modulated effects of anesthetics on neuronal pacemaker currents. J. Neurosci. 25, 5803–5814. 10.1523/JNEUROSCI.1153-05.2005 15958747 PMC6724885

[B12] Ching S. Brown E. N. (2014). Modeling the dynamical effects of anesthesia on brain circuits. Curr. Opin. Neurobiol. 25, 116–122. 10.1016/j.conb.2013.12.011 24457211 PMC4181389

[B13] Datunashvili M. Chaudhary R. Zobeiri M. Lüttjohann A. Mergia E. Baumann A.. (2018). Modulation of hyperpolarization-activated inward current and thalamic activity modes by different cyclic nucleotides. Front. Cell. Neurosci. 12:369. 10.3389/fncel.2018.00369 30405353 PMC6207575

[B14] Destexhe A. Sejnowski T. J. (2003). Interactions between membrane conductances underlying thalamocortical slow-wave oscillations. Physiol. Rev. 83, 1401–1453. 10.1152/physrev.00012.2003 14506309 PMC2927823

[B16] Eger E. I. II Fisher D. M. Dilger J. P. Sonner J. M. Evers A. Franks N. P.. (2001). Relevant concentrations of inhaled anesthetics for *in vitro* studies of anesthetic mechanisms. Anesthesiology 94, 915–921. 10.1097/00000542-200105000-00032 11388545

[B15] Eger E. I. Saidman L. J. Brandstater B. (1965). Minimum alveolar anesthetic concentration. Anesthesiology 26, 756–763. 10.1097/00000542-196511000-00010 5844267

[B17] Franks N. P. (2008). General anaesthesia: from molecular targets to neuronal pathways of sleep and arousal. Nat. Rev. Neurosci. 9, 370–386. 10.1038/nrn2372 18425091

[B18] Franks N. P. Lieb W. R. (1993). Selective actions of volatile general anaesthetics at molecular and cellular levels. Br. J. Anaesth. 71, 65–76. 10.1093/bja/71.1.65 7688242

[B19] Franks N. P. Lieb W. R. (1996). Temperature dependence of the potency of volatile general anesthetics: implications for *in vitro* experiments. Anesthesiology 84, 716–720. 10.1097/00000542-199603000-00027 8659800

[B20] Frere S. G. Luthi A. (2004). Pacemaker channels in mouse thalamocortical neurones are regulated by distinct pathways of cAMP synthesis. J. Physiol. 554, 111–125. 10.1113/jphysiol.2003.050989 14678496 PMC1664735

[B21] Garcia P. S. Kolesky S. E. Jenkins A. (2010). General anesthetic actions on GABA_A_ receptors. Curr. Neuropharmacol. 8, 2–9. 10.2174/157015910790909502 20808541 PMC2866459

[B22] Goldstein P. A. (2015). HCN1 channels as targets for volatile anesthetics: coming to the fore. Anesth. Analg. 121, 594–596. 10.1213/ANE.0000000000000871 26287292

[B23] Guillery R. W. Sherman S. (2002). Thalamic relay functions and their role in corticocortical communication. Neuron 33, 163–175. 10.1016/s0896-6273(01)00582-7 11804565

[B24] Guillery R. W. Sherman S. M. (2011). Branched thalamic afferents: what are the messages that they relay to the cortex? Brain Res. Rev. 66, 205–219. 10.1016/j.brainresrev.2010.08.001 20696186 PMC3657838

[B25] Hapfelmeier G. Schneck H. Kochs E. (2001). Sevoflurane potentiates and blocks GABA-induced currents through recombinant α1β2γ2 GABA_A_ receptors: implications for an enhanced GABAergic transmission. Eur. J. Anaesthesiol. 18, 377–383. 10.1046/j.0265-0215.2001.00848.x 11412290

[B26] Haseneder R. Kratzer S. von Meyer L. Eder M. Kochs E. Rammes G. (2009). Isoflurane and sevoflurane dose-dependently impair hippocampal long-term potentiation. Eur. J. Pharmacol. 623, 47–51. 10.1016/j.ejphar.2009.09.022 19765574

[B27] He C. Chen F. Li B. Hu Z. (2014). Neurophysiology of HCN channels: from cellular functions to multiple regulations. Prog. Neurobiol. 112, 1–23. 10.1016/j.pneurobio.2013.10.001 24184323

[B28] Hudetz A. G. Vizuete J. A. Imas O. A. (2009). Desflurane selectively suppresses long-latency cortical neuronal response to flash in the rat. Anesthesiology 111, 231–239. 10.1097/ALN.0b013e3181ab671e 19568167 PMC2761678

[B29] Kaupp U. B. Seifert R. (2001). Molecular diversity of pacemaker ion channels. Annu. Rev. Physiol. 63, 235–257. 10.1146/annurev.physiol.63.1.235 11181956

[B30] Kratzer S. Mattusch C. Garcia P. S. Schmid S. Kochs E. Rammes G.. (2017). Propofol and sevoflurane differentially modulate cortical depolarization following electric stimulation of the ventrobasal thalamus. Front. Comput. Neurosci. 11:109. 10.3389/fncom.2017.00109 29321737 PMC5732174

[B31] Kubota H. Akaike H. Okamitsu N. Jang I.-S. Nonaka K. Kotani N.. (2020). Xenon modulates the GABA and glutamate responses at genuine synaptic levels in rat spinal neurons. Brain Res. Bull. 157, 51–60. 10.1016/j.brainresbull.2020.01.016 31987927

[B32] Kuroda M. Yoshikawa D. Nishikawa K. Saito S. Goto F. (2004). Volatile anesthetics inhibit calcitonin gene-related peptide receptor-mediated responses in pithed rats and human neuroblastoma cells. J. Pharmacol. Exp. Ther. 311, 1016–1022. 10.1124/jpet.104.071936 15297469

[B33] Lewis A. S. Estep C. M. Chetkovich D. M. (2010). The fast and slow ups and downs of HCN channel regulation. Channels 4, 215–231. 10.4161/chan.4.3.11630 20305382 PMC4349509

[B34] Liu T. Petrof I. Sherman S. M. (2015). Modulatory effects of activation of metabotropic glutamate receptors on GABAergic circuits in the mouse thalamus. J. Neurophysiol. 113, 2646–2652. 10.1152/jn.01014.2014 25652932 PMC4416614

[B35] Liu X. Lauer K. K. Ward B. D. Li S. J. Hudetz A. G. (2013). Differential effects of deep sedation with propofol on the specific and nonspecific thalamocortical systems: a functional magnetic resonance imaging study. Anesthesiology 118, 59–69. 10.1097/ALN.0b013e318277a801 23221862 PMC4080838

[B36] Lörincz A. Notomi T. Tamás G. Shigemoto R. Nusser Z. (2002). Polarized and compartment-dependent distribution of HCN1 in pyramidal cell dendrites. Nat. Neurosci. 5, 1185–1193. 10.1038/nn962 12389030

[B37] Ludwig A. Budde T. Stieber J. Moosmang S. Wahl C. Holthoff K.. (2003). Absence epilepsy and sinus dysrhythmia in mice lacking the pacemaker channel HCN2. EMBO J. 22, 216–224. 10.1093/emboj/cdg032 12514127 PMC140107

[B38] Ludwig A. Zong X. Jeglitsch M. Hofmann F. Biel M. (1998). A family of hyperpolarization-activated mammalian cation channels. Nature 393, 587–591. 10.1038/31255 9634236

[B39] Ludwig A. Zong X. Stieber J. Hullin R. Hofmann F. Biel M. (1999). Two pacemaker channels from human heart with profoundly different activation kinetics. EMBO J. 18, 2323–2329. 10.1093/emboj/18.9.2323 10228147 PMC1171315

[B40] Lyashchenko A. K. Redd K. J. Yang J. Tibbs G. R. (2007). Propofol inhibits HCN1 pacemaker channels by selective association with the closed states of the membrane embedded channel core. J. Physiol. 583, 37–56. 10.1113/jphysiol.2007.136465 17569731 PMC2277223

[B41] Mashour G. A. (2014). Top-down mechanisms of anesthetic-induced unconsciousness. Front. Syst. Neurosci. 8:115. 10.3389/fnsys.2014.00115 25002838 PMC4066704

[B42] Mashour G. A. Hudetz A. G. (2017). Bottom-up and top-down mechanisms of general anesthetics modulate different dimensions of consciousness. Front. Neural Circuits 11:44. 10.3389/fncir.2017.00044 28676745 PMC5476707

[B43] Mattusch C. Kratzer S. Buerge M. Kreuzer M. Engel T. Kopp C.. (2015). Impact of hyperpolarization-activated, cyclic nucleotide-gated cation channel type 2 for the xenon-mediated anesthetic effect: evidence from *in vitro* and *in vivo* experiments. Anesthesiology 122, 1047–1059. 10.1097/ALN.0000000000000635 25782754

[B44] McCormick D. A. Bal T. (1997). Sleep and arousal: thalamocortical mechanisms. Annu. Rev. Neurosci. 20, 185–215. 10.1146/annurev.neuro.20.1.185 9056712

[B45] McCormick D. A. Pape H. C. (1990). Properties of a hyperpolarization-activated cation current and its role in rhythmic oscillation in thalamic relay neurones. J. Physiol. 431, 291–318. 10.1113/jphysiol.1990.sp018331 1712843 PMC1181775

[B46] Meuth S. G. Kanyshkova T. Meuth P. Landgraf P. Munsch T. Ludwig A.. (2006). Membrane resting potential of thalamocortical relay neurons is shaped by the interaction among TASK3 and HCN2 channels. J. Neurophysiol. 96, 1517–1529. 10.1152/jn.01212.2005 16760342

[B47] Neske G. T. (2016). The slow oscillation in cortical and thalamic networks: mechanisms and functions. Front. Neural Circuits 9:88. 10.3389/fncir.2015.00088 26834569 PMC4712264

[B48] Nishikawa K. Kubo K. Obata H. Yanagawa Y. Saito S. (2011). The influence of manipulations to alter ambient GABA concentrations on the hypnotic and immobilizing actions produced by sevoflurane, propofol and midazolam. Neuropharmacology 61, 172–180. 10.1016/j.neuropharm.2011.03.025 21497611

[B49] Notomi T. Shigemoto R. (2004). Immunohistochemical localization of I_h_ channel subunits, HCN1-4, in the rat brain. J. Comp. Neurol. 471, 241–276. 10.1002/cne.11039 14991560

[B50] Pape H. C. (1996). Queer current and pacemaker: the hyperpolarization-activated cation current in neurons. Annu. Rev. Physiol. 58, 299–327. 10.1146/annurev.ph.58.030196.001503 8815797

[B51] Ranft A. Golkowski D. Kiel T. Riedl V. Kohl P. Rohrer G.. (2016). Neural correlates of sevoflurane-induced unconsciousness identified by simultaneous functional magnetic resonance imaging and electroencephalography. Anesthesiology 125, 861–872. 10.1097/ALN.0000000000001322 27617689 PMC5069173

[B52] Raz A. Grady S. M. Krause B. M. Uhlrich D. J. Manning K. A. Banks M. I. (2014). Preferential effect of isoflurane on top-down vs. bottom-up pathways in sensory cortex. Front. Syst. Neurosci. 8:191. 10.3389/fnsys.2014.00191 25339873 PMC4188029

[B53] Riegelhaupt P. M. Tibbs G. R. Goldstein P. A. (2018). HCN and K2P Channels in Anesthetic Mechanisms Research. Methods Enzymol. 602, 391–416. 10.1016/bs.mie.2018.01.015 29588040

[B54] Ries C. R. Puil E. (1999). Mechanism of anesthesia revealed by shunting actions of isoflurane on thalamocortical neurons. J. Neurophysiol. 81, 1795–1801. 10.1152/jn.1999.81.4.1795 10200213

[B55] Robinson R. B. Siegelbaum S. A. (2003). Hyperpolarization-activated cation currents: from molecules to physiological function. Annu. Rev. Physiol. 65, 453–480. 10.1146/annurev.physiol.65.092101.142734 12471170

[B56] Rudolph U. Antkowiak B. (2004). Molecular and neuronal substrates for general anaesthetics. Nat. Rev. Neurosci. 5, 709–720. 10.1038/nrn1496 15322529

[B57] Santoro B. Chen S. Luthi A. Pavlidis P. Shumyatsky G. P. Tibbs G. R.. (2000). Molecular and functional heterogeneity of hyperpolarization-activated pacemaker channels in the mouse CNS. J. Neurosci. 20, 5264–5275. 10.1523/JNEUROSCI.20-14-05264.2000 10884310 PMC6772310

[B58] Seifert R. Scholten A. Gauss R. Mincheva A. Lichter P. Kaupp U. B. (1999). Molecular characterization of a slowly gating human hyperpolarization-activated channel predominantly expressed in thalamus, heart and testis. Proc. Natl. Acad. Sci. U S A 96, 9391–9396. 10.1073/pnas.96.16.9391 10430953 PMC17793

[B59] Sherman S. M. (2016). Thalamus plays a central role in ongoing cortical functioning. Nat. Neurosci. 19, 533–541. 10.1038/nn.4269 27021938

[B60] Sherman S. M. Guillery R. W. (1996). Functional organization of thalamocortical relays. J. Neurophysiol. 76, 1367–1395. 10.1152/jn.1996.76.3.1367 8890259

[B61] Sirois J. E. Pancrazio J. J. Lynch C. Bayliss D. A. (1998). Multiple ionic mechanisms mediate inhibition of rat motoneurones by inhalation anaesthetics. J. Physiol. 512, 851–862. 10.1111/j.1469-7793.1998.851bd.x 9769427 PMC2231236

[B62] Stieber J. Stockl G. Herrmann S. Hassfurth B. Hofmann F. (2005). Functional expression of the human HCN3 channel. J. Biol. Chem. 280, 34635–34643. 10.1074/jbc.M502508200 16043489

[B63] Sugasawa Y. Fukuda M. Ando N. Inoue R. Nakauchi S. Miura M.. (2018). Modulation of hyperpolarization-activated cation current I_h_ by volatile anesthetic sevoflurane in the mouse striatum during postnatal development. Neurosci. Res. 132, 8–16. 10.1016/j.neures.2017.09.009 28970101

[B64] van Welie I. van Hooft J. A. Wadman W. J. (2004). Homeostatic scaling of neuronal excitability by synaptic modulation of somatic hyperpolarization-activated I_h_ channels. Proc. Natl. Acad. Sci. U S A 101, 5123–5128. 10.1073/pnas.0307711101 15051886 PMC387384

[B65] Voss L. J. García P. S. Hentschke H. Banks M. I. (2019). Understanding the effects of general anesthetics on cortical network activity using *ex vivo* preparations. Anesthesiology 130, 1049–1063. 10.1097/ALN.0000000000002554 30694851 PMC6520142

[B66] Wainger B. J. DeGennaro M. Santoro B. Siegelbaum S. A. Tibbs G. R. (2001). Molecular mechanism of cAMP modulation of HCN pacemaker channels. Nature 411, 805–810. 10.1038/35081088 11459060

[B67] Wan X. Mathers D. A. Puil E. (2003). Pentobarbital modulates intrinsic and GABA-receptor conductances in thalamocortical inhibition. Neuroscience 121, 947–958. 10.1016/j.neuroscience.2003.07.002 14580945

[B68] Wang J. Chen S. Nolan M. F. Siegelbaum S. A. (2002). Activity-dependent regulation of HCN pacemaker channels by cyclic AMP: signaling through dynamic allosteric coupling. Neuron 36, 451–461. 10.1016/s0896-6273(02)00968-6 12408847

[B69] Wang J. Chen S. Siegelbaum S. A. (2001). Regulation of hyperpolarization-activated HCN channel gating and cAMP modulation due to interactions of COOH terminus and core transmembrane regions. J. Gen. Physiol. 118, 237–250. 10.1085/jgp.118.3.237 11524455 PMC2229504

[B70] Whary M. T. Baumgarth N. Fox J. G. Barthold S. W. (2015). “Biology and diseases of mice,” in Laboratory Animal Medicine, eds Fox J. G. Anderson L. C. Otto G. M. Pritchett-Corning K. R. Whary M. T. (London: Elsevier Academic Press), 43–149.

[B71] Xiong W. X. Zhou G. X. Wang B. Xue Z. G. Wang L. Sun H. C.. (2013). Impaired spatial learning and memory after sevoflurane-nitrous oxide anesthesia in aged rats is associated with down-regulated cAMP/CREB signaling. PLoS One 8:e79408. 10.1371/journal.pone.0079408 24260214 PMC3829840

[B73] Ying S.-W. Abbas S. Y. Harrison N. L. Goldstein P. A. (2006). Propofol block of I_h_ contributes to the suppression of neuronal excitability and rhythmic burst firing in thalamocortical neurons. Eur. J. Neurosci. 23, 465–480. 10.1111/j.1460-9568.2005.04587.x 16420453

[B72] Ying S. W. Goldstein P. A. (2005). Propofol-block of SK channels in reticular thalamic neurons enhances GABAergic inhibition in relay neurons. J. Neurophysiol. 93, 1935–1948. 10.1152/jn.01058.2004 15563549

[B74] Yue B. W. Huguenard J. R. (2001). The role of H-current in regulating strength and frequency of thalamic network oscillations. Thalamus Relat. Syst. 1, 95–103. 10.1016/S1472-9288(01)00009-7 18239728 PMC2222919

[B75] Zobeiri M. Chaudhary R. Blaich A. Rottmann M. Herrmann S. Meuth P.. (2019). The hyperpolarization-activated HCN4 channel is important for proper maintenance of oscillatory activity in the thalamocortical system. Cereb. Cortex 29, 2291–2304. 10.1093/cercor/bhz047 30877792 PMC6458902

[B76] Zobeiri M. Chaudhary R. Datunashvili M. Heuermann R. J. Lüttjohann A. Narayanan V.. (2017). Modulation of thalamocortical oscillations by TRIP8b, an auxiliary subunit for HCN channels. Brain Struct. Funct. 223, 1537–1564. 10.1007/s00429-017-1559-z 29168010 PMC5869905

