# Supplementary material for: Attenuation of Native Hyperpolarization-Activated, Cyclic Nucleotide-Gated Channel Function by the Volatile Anesthetic Sevoflurane in Mouse Thalamocortical Relay Neurons
Source: Front Cell Neurosci. 2021 Jan 21;14:606687. doi: 10.3389/fncel.2020.606687 (PMC7858256; doi:10.3389/fncel.2020.606687)
Supplement: Supplementary file 1 [file Table_1.DOCX]

Supplementary Material

Table S1: Effects of sevoflurane on the resting membrane potential in thalamocortical relay neurons at different concentration groups (absolute values).

|  | **resting membrane potential [mV]** | | | |  |
| --- | --- | --- | --- | --- | --- |
|  | control | | sevoflurane | |  |
| **c_aq_ sevoflurane [mM]** | *Median* | *IQR* | *Median* | *IQR* | *n* |
| **0.08** | -56.2 | -57.8 – -55.3 | -55.7 | -58.1 – -54.5 | 10 |
| **0.33** | -56.9 | -57.9 – -56.3 | -57.0 | -58.7 – -55.3 | 9 |
| **0.45** | -56.7 | -58.5 – -54.9 | -56.8 | -58.3 – -54.6 | 12 |
| **0.69** | -58.8 | -60.1 – -54.9 | -60.7 | -62.2 – -58.1 | 9 |
| **1.47** | -58.1 | -60.8 – -56.7 | -63.1 | -65.1 – -59.7 | 5 |
| **0.69 + ZD7288 (0.04 mM)** | -60.3 | -61.9 – -59.2 | -70.3 | -73.8 – -65.4 | 7 |

Table S2: Effects of sevoflurane on the input resistance in thalamocortical relay neurons at different concentration groups (absolute values).

|  | **input resistance [MΩ]** | | | |  |
| --- | --- | --- | --- | --- | --- |
|  | control | | sevoflurane | |  |
| **c_aq_ sevoflurane [mM]** | *Median* | *IQR* | *Median* | *IQR* | *n* |
| **0.08** | 210.6 | 163.2–267.4 | 187.5 | 149.5–239.7 | 10 |
| **0.33** | 198.3 | 168.7–232.8 | 150.7 | 129.8–164.1 | 9 |
| **0.45** | 227.8 | 199.8–262.0 | 190.3 | 132.4–220.7 | 12 |
| **0.69** | 297.6 | 222.2–373.4 | 160.6 | 128.5–182.0 | 9 |
| **1.47** | 272.4 | 213.5–306.9 | 148.1 | 84.0–150.9 | 5 |
| **0.69 + ZD7288 (0.04 mM)** | 276.7 | 200.3–348.6 | 434.7 | 163.5–600.3 | 7 |

Table S3: Effects of sevoflurane on the action potential threshold in thalamocortical relay neurons at different concentration groups (absolute values).

|  | **action potential threshold [mV]** | | | |  |
| --- | --- | --- | --- | --- | --- |
|  | control | | sevoflurane | |  |
| **c_aq_ sevoflurane [mM]** | *Median* | *IQR* | *Median* | *IQR* | *n* |
| **0.08** | -38.6 | -39.9 – -36.4 | -37.5 | -39.1 – -35.4 | 10 |
| **0.33** | -36.5 | -38.9 – -35.4 | -35.3 | -36.9 – -32.9 | 9 |
| **0.45** | -38.6 | -40.1 – -34.1 | -37.0 | -39.7 – -32.4 | 12 |
| **0.69** | -40.6 | -42.0 – -37.6 | -38.0 | -40.5 – -36.7 | 9 |
| **1.47** | -39.7 | -42.3 – -37.7 | -36.7 | -39.7 – -34.9 | 5 |

Table S4: Effects of sevoflurane on the action potential frequency in thalamocortical relay neurons at different concentration groups (absolute values).

|  | **action potential frequency [Hz]** | | | |  |
| --- | --- | --- | --- | --- | --- |
|  | control | | sevoflurane | |  |
| **c_aq_ sevoflurane [mM]** | *Median* | *IQR* | *Median* | *IQR* | *n* |
| **0.08** | 41.3 | 39.4–45.6 | 44.2 | 38.3–48.1 | 10 |
| **0.33** | 41.7 | 35.8–43.3 | 37.5 | 32.9–42.9 | 9 |
| **0.45** | 37.5 | 35.0–46.1 | 30.8 | 27.1–43.3 | 12 |
| **0.69** | 31.7 | 28.3–41.7 | 26.7 | 22.5–34.6 | 9 |
| **1.47** | 33.3 | 25.4–43.9 | 18.3 | 7.6–29.6 | 5 |

Table S5: Effects of sevoflurane on I_h maximal_ (recorded at ‑133 mV) in thalamocortical relay neurons at different concentration groups (absolute values).

|  | **I_h maximal_ [pA]** | | | |  |
| --- | --- | --- | --- | --- | --- |
|  | control | | sevoflurane | |  |
| **c_aq_ sevoflurane [mM]** | *Median* | *IQR* | *Median* | *IQR* | *n* |
| **0.08** | 1050.5 | 671.2–1317.2 | 1089.6 | 764.1–1335.9 | 10 |
| **0.33** | 1176.1 | 1006.4–1595.0 | 1183.3 | 917.1– 1432.0 | 9 |
| **0.45** | 1038.8 | 873.2–1185.2 | 949.2 | 748.4–1053.0 | 12 |
| **0.69** | 838.4 | 530.5–1065.1 | 444.6 | 351.4–621.7 | 15 |
| **1.47** | 743.83 | 637.6–1137.3 | 279.37 | 241.9–631.5 | 5 |
| **0.69 + ZD7288 (0.04 mM)** | 800.3 | 500.5–939.5 | 74.1 | 49.2–92.8 | 7 |

Table S6: Effects of sevoflurane on the half-maximal activation potential of HCN channels in thalamocortical relay neurons at different concentration groups (absolute values).

|  | **half-maximal activation potential [mV]** | | | |  |
| --- | --- | --- | --- | --- | --- |
|  | control | | sevoflurane | |  |
| **c_aq_ sevoflurane [mM]** | *Median* | *IQR* | *Median* | *IQR* | *n* |
| **0.08** | -84.2 | -86.4 – -82.3 | -83.4 | -84.2 – -82.3 | 10 |
| **0.33** | -84.9 | -86.5 – -82.5 | -82.0 | -83.8 – -80.1 | 9 |
| **0.45** | -85.0 | -85.7 – -83.4 | -83.7 | -89.1 – 82.0 | 12 |
| **0.69** | -88.4 | -90.1 – -86.9 | -89.5 | -92.9 – -87.6 | 15 |
| **1.47** | -86.2 | -89.9 – -85.6 | -94.8 | -98.0 – -92.0 | 5 |

Table S7: Effects of sevoflurane on the HCN activation time constant τ_fast_ in thalamocortical relay neurons at different concentration groups (absolute values).

|  | **τ_fast_ [ms]** | | | |  |
| --- | --- | --- | --- | --- | --- |
|  | control | | sevoflurane | |  |
| **c_aq_ sevoflurane [mM]** | *Median* | *IQR* | *Median* | *IQR* | *n* |
| **0.08** | 190 | 166–243 | 216 | 178–244 | 10 |
| **0.33** | 202 | 179–229 | 200 | 185–230 | 9 |
| **0.45** | 212 | 176–252 | 217 | 188–255 | 12 |
| **0.69** | 257 | 219–294 | 330 | 270–371 | 9 |
| **1.47** | 264 | 171–273 | 399 | 368–605 | 5 |

Table S8: Effects of sevoflurane on the HCN activation time constant τ_slow_ in thalamocortical relay neurons at different concentration groups (absolute values).

|  | **τ_slow_ [ms]** | | | |  |
| --- | --- | --- | --- | --- | --- |
|  | control | | sevoflurane | |  |
| **c_aq_ sevoflurane [mM]** | *Median* | *IQR* | *Median* | *IQR* | *n* |
| **0.08** | 1008 | 850–1111 | 1083 | 730–1273 | 10 |
| **0.33** | 1268 | 1039–1477 | 1296 | 1229–1399 | 9 |
| **0.45** | 997 | 895–1253 | 1091 | 872–1326 | 12 |
| **0.69** | 1200 | 1057–2076 | 1262 | 1075–1710 | 9 |
| **1.47** | 1189 | 1055–1924 | 1513 | 1243–1691 | 5 |

Table S9: Effects of sevoflurane on the voltage sag amplitude in thalamocortical relay neurons at different concentration groups (absolute values).

|  | **voltage sag amplitude [mV]** | | | |  |
| --- | --- | --- | --- | --- | --- |
|  | control | | sevoflurane | |  |
| **c_aq_ sevoflurane [mM]** | *Median* | *IQR* | *Median* | *IQR* | *n* |
| **0.08** | 54.6 | 42.2–74.8 | 56.8 | 35.8–65.2 | 10 |
| **0.33** | 51.9 | 45.5–64.9 | 37.1 | 31.9–43.2 | 9 |
| **0.45** | 57.0 | 53.4–62.8 | 43.0 | 22.3–61.8 | 12 |
| **0.69** | 67.6 | 50.1–88.0 | 39.8 | 22.0–56.9 | 9 |
| **1.47** | 63.5 | 52.6–74.3 | 17.3 | 9.2–33.4 | 5 |

Table S10: Effects of sevoflurane on the rebound burst delay in thalamocortical relay neurons at different concentration groups (absolute values).

|  | **rebound burst delay [ms]** | | | |  |
| --- | --- | --- | --- | --- | --- |
|  | control | | sevoflurane | |  |
| **c_aq_ sevoflurane [mM]** | *Median* | *IQR* | *Median* | *IQR* | *n* |
| **0.08** | 28.9 | 25.3–33.9 | 31.9 | 25.1–36.6 | 10 |
| **0.33** | 30.8 | 28.3–33.0 | 39.0 | 33.1–44.6 | 9 |
| **0.45** | 32.9 | 29.3–36.8 | 39.8 | 33.5–47.9 | 12 |
| **0.69** | 29.0 | 24.8–30.5 | 46.8 | 39.6–54.0 | 9 |
| **1.47** | 29.5 | 26.0–36.0 | 71.3 | 57.4–94.8 | 5 |

Table S11: Effects of sevoflurane on the number of action potentials during rebound bursts in thalamocortical relay neurons at different concentration groups (absolute values).

|  | **number of action potentials (n)** | | | |  |
| --- | --- | --- | --- | --- | --- |
|  | control | | sevoflurane | |  |
| **c_aq_ sevoflurane [mM]** | *Median* | *IQR* | *Median* | *IQR* | *n* |
| **0.08** | 7.0 | 5.8–8.3 | 6.5 | 5.5–8.0 | 10 |
| **0.33** | 6.0 | 5.0–7.5 | 4.0 | 3.0–4.0 | 9 |
| **0.45** | 6.5 | 5.3–10.0 | 4.0 | 3.0–4.8 | 12 |
| **0.69** | 6.0 | 4.5–13.5 | 2.0 | 1.0–2.0 | 9 |
| **1.47** | 8.0 | 5.5–10.5 | 0 | 0–1.0 | 5 |

Table S12: Effects of sevoflurane on the rebound burst duration in thalamocortical relay neurons at different concentration groups (absolute values).

|  | **rebound burst duration [ms]** | | | |  |
| --- | --- | --- | --- | --- | --- |
|  | control | | sevoflurane | |  |
| **c_aq_ sevoflurane [mM]** | *Median* | *IQR* | *Median* | *IQR* | *n* |
| **0.08** | 73.3 | 53.5–138.8 | 66.3 | 47.5–96.6 | 10 |
| **0.33** | 65.0 | 57.0–103.3 | 32.0 | 21.3–41.3 | 9 |
| **0.45** | 75.8 | 50.4–144.4 | 33.8 | 19.9–48.5 | 12 |
| **0.69** | 72.5 | 50.3–180.0 | 15.5 | 11.8–17.0 | 9 |
| **1.47** | 124.0 | 71.5–207.8 | 11.0 | 9.5–12.0 | 5 |
